# Maternal fiber-rich diet promotes early-life intestinal development in offspring through milk-derived extracellular vesicles carrying miR-146a-5p

**DOI:** 10.1186/s12951-024-02344-4

**Published:** 2024-02-16

**Authors:** Dongdong Lu, Yisi Liu, Luyuan Kang, Xiangyu Zhang, Jie Hu, Hao Ye, Bingxu Huang, Yujun Wu, Jinbiao Zhao, Zhaolai Dai, Junjun Wang, Dandan Han

**Affiliations:** 1 State Key Laboratory of Animal Nutrition and Feeding, College of Animal Science and Technology, China Agricultural University, Beijing, 100193 China; 2 Adaptation Physiology Group, Wageningen University & Research, Wageningen, 6700 AH The Netherlands

**Keywords:** Maternal diet, Resistant starch, Offspring, Intestinal development, Milk-derived extracellular vesicles, miR-146a-5p

## Abstract

**Backgrounds:**

The intestinal development in early life is profoundly influenced by multiple biological components of breast milk, in which milk-derived extracellular vesicles (mEVs) contain a large amount of vertically transmitted signal from the mother. However, little is known about how maternal fiber-rich diet regulates offspring intestinal development by influencing the mEVs.

**Results:**

In this study, we found that maternal resistant starch (RS) consumption during late gestation and lactation improved the growth and intestinal health of offspring. The mEVs in breast milk are the primary factor driving these beneficial effects, especially enhancing intestinal cell proliferation and migration. To be specific, administration of mEVs after maternal RS intake enhanced intestinal cell proliferation and migration in vivo (performed in mice model and indicated by intestinal histological observation, EdU assay, and the quantification of cyclin proteins) and in vitro (indicated by CCK8, MTT, EdU, and wound healing experiments). Noteworthily, miR-146a-5p was found to be highly expressed in the mEVs from maternal RS group, which also promotes intestinal cell proliferation in cells and mice models. Mechanically, miR-146a-5p target to silence the expression of ubiquitin ligase 3 gene NEDD4L, thereby inhibiting DVL2 ubiquitination, activating the Wnt pathway, and promoting intestinal development.

**Conclusion:**

These findings demonstrated the beneficial role of mEVs in the connection between maternal fiber rich diet and offspring intestinal growth. In addition, we identified a novel miRNA-146a-5p-NEDD4L-β-catenin/Wnt signaling axis in regulating early intestinal development. This work provided a new perspective for studying the influence of maternal diet on offspring development.

**Graphical Abstract:**

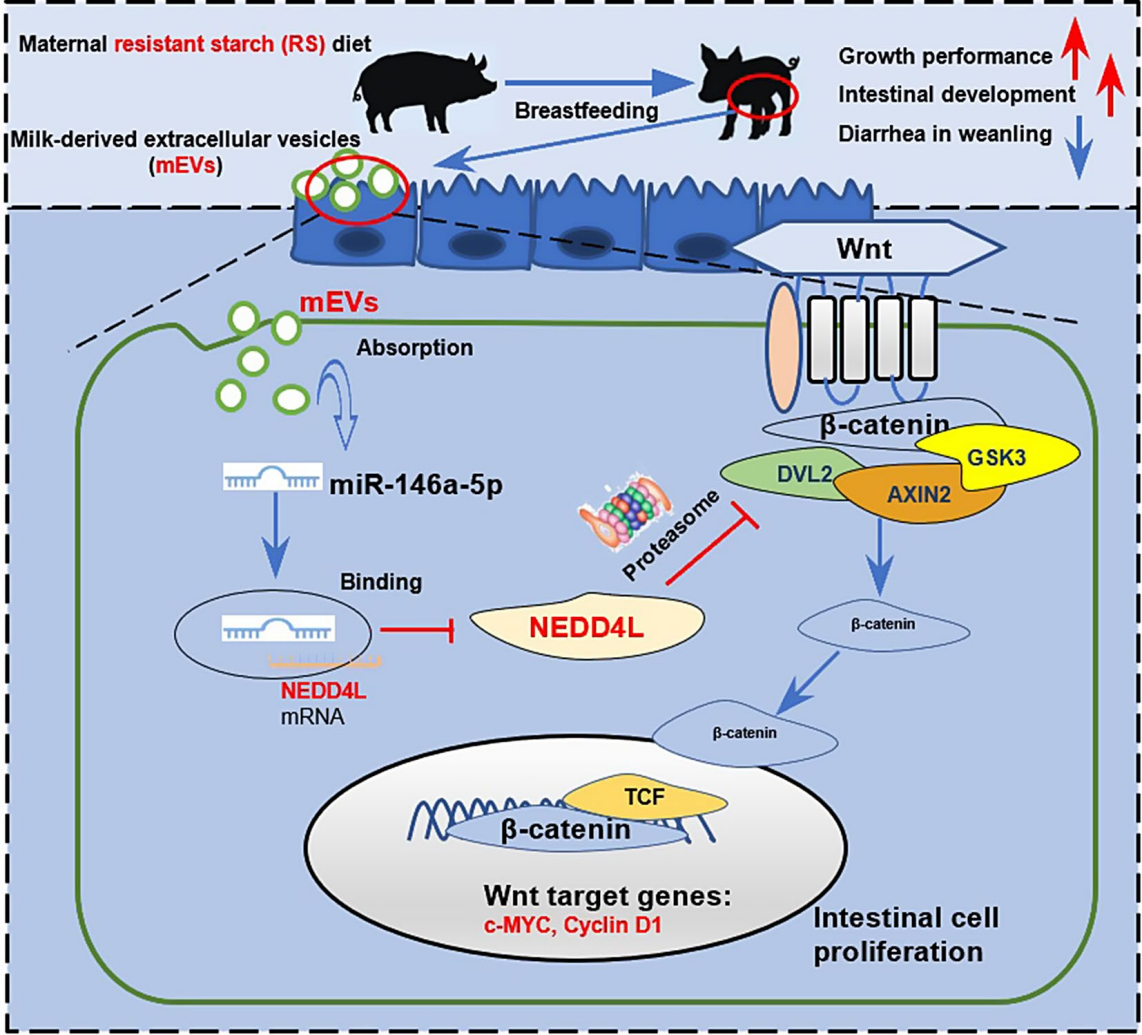

**Supplementary Information:**

The online version contains supplementary material available at 10.1186/s12951-024-02344-4.

## Introduction

As the first physiological step of transporting nutrients to the host cells, the gastrointestinal tract affects the nutrient absorption and metabolic activities of the body [1]. Special attention needs to be paid to the early intestinal development of newborn infants, since the functions of their gastrointestinal tracts are under development and which require external nutritional interventions for healthy growth [2]. Therefore, an adequate and reasonable nutrient supply is very important for the early intestinal development of animals. Breastfeeding, as a vertical transmission method from the mother, is also an important nutrition source for infants. Breast milk contains lots of maternal bioactive components, such as growth factors, chemokines, cytokines, and immunoglobulins (IgA, IgM, and IgG), that are beneficial to the postnatal gastrointestinal tract development and immunity homeostasis in newborns [3, 4].

The milk-derived extracellular vesicles (mEVs), an enriched component discovered in breast milk recently, has been emergingly proved to be crucial in regulating intestinal barrier function and promoting neonate growth, both for human and animals [5–7]. mEVs can transfer their bioactive components (such as mRNA, miRNA, DNA, proteins, and lipids) to neonates, being an important biological bond between mothers and their offspring [8–10]. Evidence also suggested that miRNAs in mEVs are the main cargos causing intestinal alterations in infants [11, 12]. When miRNAs enter the intestine cells with the absorption of mEVs, it will regulate the gene expression and physiological activities of cells, thereby promoting intestinal development and the immune function of infants [13–16]. For example, miR-34a in yak mEVs promotes the growth of intestinal epithelial cells against injury in a hypoxic environment [17]. miR-221/222 from mEVs can reverse the deoxynivalenol-induced apoptosis and growth inhibition in intestinal epithelial cells by silencing the gene of PTEN [18]. Moreover, miRNAs in mEVs can prevent intestinal epithelium disruption and inflammation by regulating intestinal immunity [1, 19]. It indicates that mEVs-derived miRNAs may serve as a key regulator during offspring intestinal health [20].

The biological information in mEVs is mainly determined by the mother. For instance, miRNAs profiles in mEVs are not only correlated with maternal body condition [21–23], but also strongly related to maternal nutritional metabolism and dietary structure [24–26]. Moreover, a recent study indicated nearly half of the microRNAs in breast milk could be affected by maternal dietary fiber sources [27]. This indicates the possibility of manipulating mEVs information by altering the maternal diet and subsequently promoting the intestinal development of offspring. This hypothesis was also confirmed by the study from Quan et al. that the supplementation of fiber in the maternal diet could affect mEVs-derived miRNAs that are potentially involved in energy metabolism and nutrient absorption of offspring [25]. Resistant starch (RS) has been proposed as a promising dietary fiber source in human and animal food. Maternal RS intake could not only benefit the body condition of mothers, but also reduce the risk of allergic disease, and improve the insulin sensitivity and glucose balance of infants [28–31]. Moreover, maternal dietary RS intake promotes the expression of mucous membrane-binding protein and improves the intestinal health of infants [32]. However, the mechanism by which maternal RS promotes intestinal health in offspring is still inconclusive, and the underlying mechanism, whether breast mEVs plays a role here, needs to be further explored.

This study, therefore, aims to investigate how maternal dietary RS supplements affect the intestinal development of offspring through mEVs. The effects of maternal RS-rich mEVs on the proliferation and renewal of offspring intestinal development were studied both in vivo and in vitro. Furthermore, a key miRNA in mEVs that mediates those processes was identified and its underlying target pathway was also demonstrated in this study. This provided a new perspective on how maternal diet influences offspring development and health.

## Methods

### Animal feeding experiment

Animal designs used in this study were approved by the animal welfare and use committee of China Agricultural University. In this study, a total of 40 sows were allocated into two groups based on their body weight and parity. During the whole experimental period (from 104 days of gestation until weaning), the control (*n* = 20) was fed with a basal diet (CON) and the treatment (*n* = 20) was fed a diet with 2% wheat bran replaced by RS fiber (RS). The total dietary fiber content was similar in the two groups and all diets were designed to meet the nutrient standards of NRC (2012) for sows (Additional file 1: Table S1). During the gestating period, sows were kept in an individual gestating stall, and fed three times per day. Before delivery, all sows were transported to the farrowing room and housed in a separate farrowing stall. Before suckling, piglets from two treatments were mixed and evenly reassigned to each lactating sow. It was ensured that the average weight and sex of piglets were the same in each group. Milk is the only food source for piglets during lactation. The weight of all piglets was recorded once a week and the weaning happened on day 21 of lactation. The diarrhea rate, diarrhea index, and health status of piglets in each litter were recorded 5 days after weaning.

### Breast mEVs isolation and EVs-deleted supernatant acquirement

Raw milk was obtained from two groups of sows during lactation. To avoid bacterial contamination, the potassium permanganate solution was used to sterilize the breast before milk collection. Raw breast milk was centrifuged at 3,000× g and 4 °C for 10 min by Eppendorf Centrifuge 5804R (Eppendorf, Hamburg, Germany) to remove cells and fats. After that, the liquids were transferred to a new tube and centrifuged at 12,000× g again to wipe off the remaining fats and cells. Next, the clean supernatant was centrifuged through ultracentrifugation (about 150,000× g) by using Optima XPN-100 Ultracentrifuge (Beckman, CA, USA) at 4 °C for 90 min. After that, the liquid was collected as the EVs-deleted supernatant. The pellet was suspended with PBS, then put into a 100 kDa filter and centrifuged for 30 min at 5,000× g. The filtered liquor was centrifuged again by ultracentrifugation at 150,000× g (90 min) and resuspended with PBS. The concentration of mEVs protein was determined using a BCA kit (Thermo Scientific, MA, USA). The morphological characteristics of mEVs were observed by Transmission electron microscopy (TEM) (Hitachi, Tokyo, Japan). The grain-diameter ratio and particle concentration of the mEVs were determined by using Nanoparticle tracking analysis (NTA) (Particle Metrix, Meerbusch, Germany). The marker proteins of mEVs (the positive CD63 and CD9, and negative Calnexin) were detected by Western blotting.

### RNA extraction and miRNA sequencing

RNA from all kinds of samples (mEVs, tissues, or cells) was extracted by using an RNA pure extraction kit (Aidlid, Beijing, China). For miRNA sequencing, the miRNA library was constructed by the Small RNA Prep Kit (Illumina, California, USA). Next, the profiles of miRNA in mEVs were obtained by using RNA sequencing which was provided by Shanghai Majorbio company, China. The filtered reads were acquired by the quality control and adaptor sequences removing on the bases of raw reads, then were matched to the Sus scrofa genome, and the numbers of miRNAs were quantified.

### The miRNA validation and mRNA expression via qRT‑PCR

For miRNA quantification, a miRNA first-strand synthesis kit (Takara, Japan) was used for the reverse transcription of miRNA, and then a SYBR premix kit (Takara, Japan) was applied in the qPCR detection. Specific primers for miRNA and U6 were provided by Sangon Biotech (Shanghai, China). The expressions of miRNAs and mRNAs were normalized to the reference genes U6 and GAPDH, respectively. The primer sequences used in this study were presented in Additional file 1: Table S2.

### Cell culture and miRNAs transfection

The porcine small intestinal epithelial cell line (IPEC-J2) and the Hep3B and embryonic kidney cell line (HEK293) used in this study were acquired from the DSMZ (Germany). After recovery, cells (4 × 10^4^ cells/mL suspension) were cultured at a condition of 37 °C, 5% CO_2_ in a DMEM medium which was added with 10% fetal bovine serum and 1% penicillin-streptomycin. RNA oligonucleotides, used in transfection experiments, were obtained from Sangon Biotech (Shanghai, China). MiRNA mimics (50 nM) or miRNA inhibitors (100 nM) were transfected into cells and two nonsense scrambled miRNA sequence was regarded as the mimic-NC and inhibitor-NC with the help of Lipofectamine 2000 (Invitrogen, MA, USA). The relative mRNA expression or relative protein levels then were detected 24 to 48 h later. For plasmid transfection, a total of 4 µg plasmid DNA was used in every 6-well plate. In the co-transfection system, cells were co-transfected with 25 nM mimics/100 nM inhibitor and 2 µg plasmid in a 6-well plate.

### The target analysis of miRNA and luciferase reporter assays

MiRanda (http://www.miranda.org) and Hybrid (bibiserv.cebitec.uni-bielefeld.de/rnahybrid) were used to scan the 3′-UTRs of mRNAs, to predict the target genes of miRNAs. For luciferase assay experiments, the potential binding position of the target sequences (or the mutant sequences) of miR-146a-5p in NEDD4L 3′-UTRs were chemically synthesized by Tsingke Biotech (Beijing, China) and then cloned into the expression vector pmirGLO (Promega, WI, USA). Next, HEK293 was transfected with miR-146a-5p mimics/mimics-NC (10 pmol) and reconstructed luciferase vector (10 ng) per well in a 96-well plate. HEK293 cells were lysed 48 h later and the fluorescence intensities of cells were determined by luciferase reporter kits (Promega, WI, USA). The intensity of Firefly was normalized to the intensity of Renilla.

### In vitro absorption of breast mEVs

To track the mEVs, CmEVs, and RmEVs were labeled by PKH26 red fluorescent dye (Bestbio, Beijing, China) according to the instructions. After washing with PBS, the CmEVs and RmEVs were suspended in 500 µL PBS with 2µL PKH26 and incubated at 37 ℃, and then centrifugated at 150,000× g. Next, the PKH26-labeled RmEVs and CmEVs were resuspended in PBS after removing the supernatant. In the in vitro absorption experiments, 1 × 10^4^ IPEC-J2 cells were incubated with 1 mg of PKH26-labeled CmEVs, RmEVs, or PBS for 30 min at 37 ℃, 5% CO_2_. After two washes by PBS, the IPEC-J2 was then dyed with a DAPI for labeling the cells. After that, the uptake of mEVs by IPEC-J2 was visualized using a confocal microscope.

### Intestinal cell proliferation and migration

The experiment of cell proliferation assay was performed following previous studies [33, 34]. In brief, 200µL IPEC-J2 cells (4 × 10^4^ cells/mL suspension) for each well were seeded in a 96-well plate. The mEVs or RNA oligonucleotides were added or transfected into IPEC-J2 24 h later and incubated for another 24 h. The viabilities of IPEC-J2 were detected by MTT and CCK8 cell counting kits (Med Chem Expression, NJ, USA). For cell migration, about 2 mL IPEC-J2 cells (4 × 10^4^ cells/mL suspension) were seeded into a 6-well plate for each well and cultured for 24 h. The cells then were treated with mEVs or RNA oligonucleotide for 24 h. Before scratching, 2 µg/mL mitomycin C (Med Chem Expression, NJ, USA) was added to inhibit cell growth. The Straight scratches in the cell plate were made by pipette tip. The images of scratch recovery were obtained at 0 h, 12 h, and 24 after scratching by microscopy (Zeiss, Oberkochen, Germany). The cell migration rate of IPEC-J2 was indicated by the recovery of scratch surface area.

### Construction of protein overexpression plasmid and tag plasmid

The over-expressing plasmid pcDNA3.1 was bought from Tsingke Biotech (Beijing, China). NEDD4L, DVL2, and Ubiquitin were amplified from the mRNA of IPEC-J2, respectively, followed by a reverse transcription reaction. The NEDD4L, DVL2, and Ubiquitin cDNA was then cloned into the expression vectors pcDNA3.1-Flag, pCMV-Myc, and pCMV-HA respectively, to construct Flag-NEDD4L, Myc-DVL2, or HA-UB expressing plasmids. All plasmids were confirmed by sequencing service from Tsingke Biotechnology Co., Ltd (Beijing, China).

### TOP/FOP flash assay

IPEC-J2 cells (4 × 10^4^ cells/mL suspension) were plated in a 96-well plate for 24 h and transfected with 100 ng reporter plasmid TOP or FOP (Beyotime Biotechnology, Shanghai, China) and 10 ng pRL-TK/Renilla (Beyotime Biotechnology, Shanghai, China) for each well by Lipofectamine 2000 (Invitrogen, MA, USA). For NEDD4L and DVL2 evaluation, the 100 ng of additional plasmid (pcDNA-NEDD4L and pcDNA-DVL2) was co-transfected in each well when indicated. For miRNA evaluation, the mimics and mimics-NC were additionally transfected into corresponding wells. The luciferase intensities were observed by the commercial Dual-Glo Luciferase Assay Kits (Promega, WI, USA) 24 h later. The intensities of TOPFlash and FOPFlash were normalized to Renilla.

### In vivo agomiRNA injection experiments and the track of Cy3-labeled agomiRNAs

For in vivo assays, 21-days-old weanling C57BL/6 mice (female, *n* = 8) were administrated with 2 nmol/10 g agomiR-146a-5p or scrambled negative control (agomiR-NC) respectively by intraperitoneal injection on day 0, 3, 6, 9, 12. Mice in the Control were administrated with PBS. Blood samples were collected for serum parameter detection. Jejunum tissues were stored at -80 ℃ or fixed by 10% formalin. For the miRNAs tracking experiments, the Cy3-labeled agomiR-146a-5p and Cy3-labeled agomiR-NC were acquired from Sangon Biotech (Shanghai, China). The mice were intraperitoneally injected with Cy3-labeled agomiR-146a-5p, Cy3-labeled agomiR-NC, and PBS, the jejunum was collected after 6 h later and stored in liquid nitrogen for section observation. DAPI staining was performed in the jejunum frozen section. After that, the sections were visualized by a confocal microscope.

### Histological observation and immunohistochemistry (IHC)

For histological analysis and IHC observation of the small intestine, the same proximal part of the small intestine from all groups was used throughout the study. The jejunum was fixed by 10% formalin and embedded by paraffin. Then, the fixed jejunum was cut into sections and spread on a slide, then deparaffinized and dyed with hematoxylin and eosin (HE). Images of the jejunum section were acquired by a microscope and analyzed by Image J. The processes of Immunohistochemistry were described by our previous study [35]. Briefly, sections were deparaffinized with the xylene and an ethanol gradient (75%, 95%, and 100%), and then washed with distilled water and steamed in a citrate buffer for 30 min for antigen retrieval. The repaired slides were subsequently subjected to incubation with primary antibody and secondary antibody.

### Ubiquitination assay

MiR-146a-5p mimics or mimics-NC, and plasmids pcDNA 3.1-NEDD3L or its control pcDNA3.1 were transfected into IPEC-J2 cells. After 42 h of incubation, 20 µM cell-permeable proteasome inhibitor MG132 (Med Chem Expression, NJ, USA) was added to cell cultures and cultured for 6 h. Next, the cells were washed three times with PBS and were lysed by a lysis buffer for 30 min for protein extraction. The protein levels were quantified using a Western blotting assay by corresponding antibodies.

### Co-immunoprecipitation and Western blot analysis

The co-immunoprecipitation assays were performed by using the Co-IP kits PK10008 (Proteintech, IL, USA) according to manufacture protocols. Briefly, cells were lysed by RAPI lysis buffer (add protease inhibitor to 1×), and the suspension was placed on ice for lysis for 30 min, reversed every 10 min during this period. Then, the lysate was centrifugated at 4 °C at 10,000 × g for 10–15 min, and the supernatant was collected for further analysis. The protein concentration of the supernatant was measured by a BCA assay kit (Thermo Fisher Scientific, MA, USA). Next, 1–3 mg total of protein from cell lysates were put into a Spin column and incubated with 1–4 ug specific antibody (Proteintech, IL, USA) or same efficiency mouse IgG (Proteintech, IL, USA) and 150–300 µL incubation buffer (supplied by PK10008 Co-IP kit) at 4 °C for 2–4 h. Then, 50 µL suspended Protein A/G beads slurry (supplied by PK10008 Co-IP kit) was added into Spin columns and rotated at 4 °C for 1–4 h. The supernatant then was drained naturally, and the precipitation complex was washed with 800 µL Washing buffer (supplied by PK10008 Co-IP kit) about 4–5 times, and the washing solution was drained naturally and discarded. Repeat the washing. The spin column subsequently was placed into a tube and centrifuged at 500× g and 4 °C for 30 s to ensure all washing buffer flowed out. Next, the spin column was transferred into a new tube to collect the elution products. The precipitation complex was eluted by 40 µL elution buffer and was left to room temperature for 5–10 min. Next, the mixture was centrifuged at 10,000× g for 1 min to collect the elution products. This step was repeated and two elutions were merged and collected. 10 µL alkali neutralization buffer (supplied by PK10008 Co-IP kit) was added to all elution products. The immunoprecipitated samples and other primordial protein lysates were analyzed by a Simple Western assay with the WES™ system (ProteinSimple, CA, USA) according to the manufacturer’s protocol [36]. Briefly, cell lysates were diluted to a final concentration of 1.0 mg/mL and loaded in triplicate into a microplate (obtained from Wes Separation module commercial assay kits, SM-W004, proteinSimple, CA, USA), along with specific primary antibodies diluted 1:300. Then the loaded microplate and corresponding separation module were then placed into the automated Western Blotting machine (ProteinSimple, CA, USA) for analyzing. Anti-HA target antibody was used to evaluate the ubiquitination of DVL2 protein in the immunoprecipitated samples. The antibodies used to detect protein expression in this study are shown in Additional file 1: Table S3. The peak area of the chemiluminescence electropherogram for each protein via detected by the Compass for SW software (ProteinSimple, CA, USA). Finally, the relative level of protein in each sample was calculated through normalization to the peak area of the referred protein β-actin. The antibodies were used only if their correct bands could be detected in this system.

### Statistical analysis

All the data analysis in this study was performed in SPSS 20.0 (SPSS Inc., IL, USA). Chi-square analysis was used to determine the diarrhea incidence of piglets. The Two-tailed unpaired t-test was applied to the comparison of two groups and the one-way ANOVA with Dunnett’s test was used for the comparison of three or more groups. Repeated measures ANOVA was performed on the data at continuous time points, followed by within-group comparisons. Univariate variance is used when the spherical hypothesis is satisfied, and multivariate variance is used otherwise. The differences between the groups at each time point were analyzed separately when the interactions were present.

## Results

### Maternal RS consumption during late gestation and lactation improves the growth and health of offspring

**Fig. 1:**
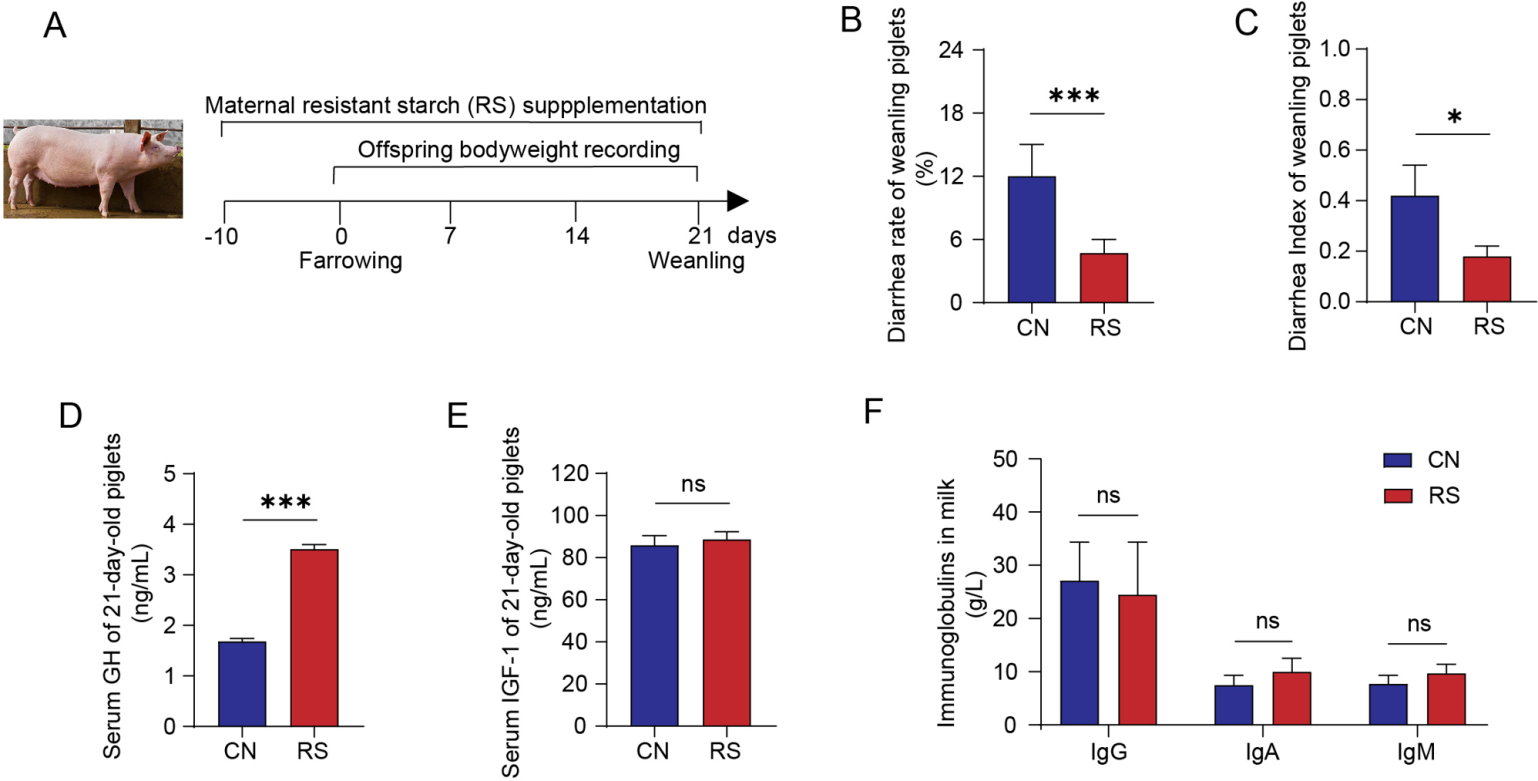
Maternal RS supplementation enhances growth performance and reduces diarrhea in weanling piglets (*n* = 20). (**A**) Animal design and maternal RS diet treatment. (**B**) Diarrhea rate of piglets during weaning. Pearson chi-square value = 11.38 (*p* < 0.001). (**C**) Diarrhea index of piglets during weaning. (**D**) Serum growth hormone (GH) level in piglets at 21 d of age. (**E**) Serum insulin-like growth factor 1 (IGF-1) level in piglets at 21 d of age. (**F**) Immunoglobulins G, A, and M in sow milk on day 21 of lactation. Data are expressed as means ± SEM. *p < 0.05, **p < 0.01, ***p < 0.001; ns, not significant

To investigate the contribution of maternal dietary manipulation on the growth of offspring, we established a maternal fiber-rich diet model from the late pregnancy of the mother to the end of lactation. Dietary 2% RS supplement beginning from day 104 of pregnancy to day 21 of lactation **(**Fig. 1A**)**. The body weight of piglets was recorded throughout the whole lactation. We found that maternal dietary RS supplementation increased litter weight, average weight, and average daily weight gain (ADG) of their piglets on day 14 and day 21 (Additional file 1 Table S4). Besides, maternal dietary RS supplementation decreased the diarrhea rate and diarrhea index of weanling piglets (Fig. 1B and C). The serum level of growth hormone (GH) was profoundly increased in piglets of RS (Fig. 1D), but not in insulin-like growth factor 1 (IGF-1) (Fig. 1E). To further investigate the maternal RS intake affects the growth of piglets through affecting the conventional components of milk, we tested the milk routine indexes and immune protein content. As presented in Fig. 1F and Additional file 1: Table S5, milk fat, protein, and immunoglobulin A, M, and G in milk were not affected by maternal RS consumption. These data indicated that maternal RS supplementation can promote growth performance and reduce the diarrhea rate of weanling piglets, but these effects are not achieved by affecting the routine nutrients and immunoglobulins in breast milk.

### mEVs from maternal RS intake mother promote intestinal development ***in vivo and in vitro***

Given that the routine nutrients and immunoglobulins in breast milk were not affected by maternal RS supplementation, the small particle mEVs from the RS group (RmEVs) and CN group (CmEVs) were extracted and analyzed. As shown in Additional file 1: Figure S1A, mEVs exhibited typical intact cup-shaped membrane vesicles based on the bilayer structure and size revealed by TEM. The diameter of mEVs ranged from 30 to 300 nm, with an average of 110 nm, which was revealed by NTA (Additional file 1: Figure S1B). To further characterize the mEVs we prepared, EVs-specific markers CD63 and CD9 were confirmed to be expressed in the mEVs by Western blotting (Additional file 1: Figure S1C). As shown in Additional file 1: Figure S1D, there are no significant differences in particle concentration between CmEVs and RmEVs (2.71 × 10^11^ particles/mL versus 2.85 × 10^11^ particles/mL of milk). Consistent with the results of marker proteins level, the mEVs-removed supernatant had a very low concentration of mEVs particles (Additional file 1: Figure S1D).

**Fig. 2:**
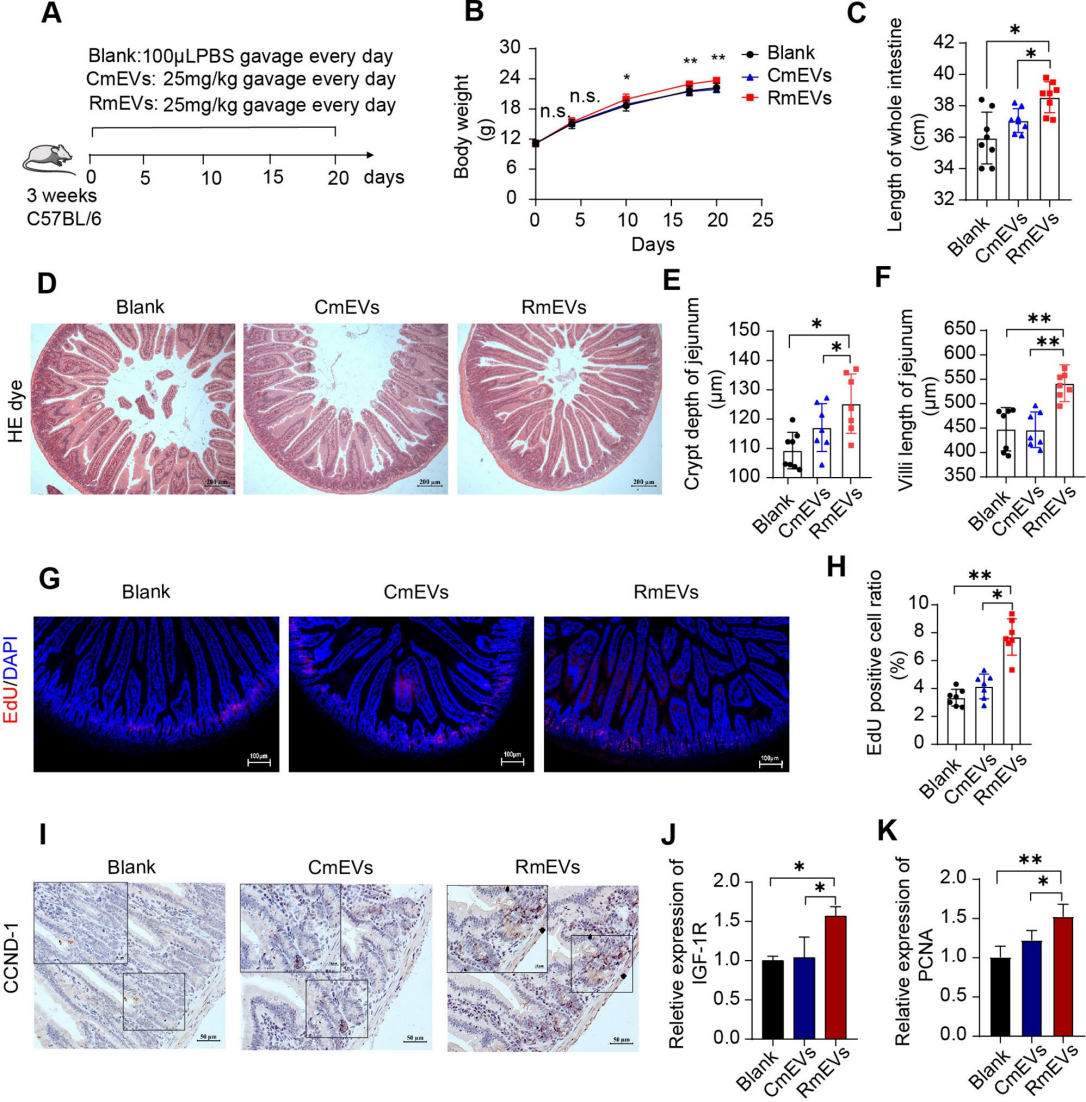
RmEVs improve intestinal development in vivo during early life (*n* = 7). (**A**) Animal designs and mEVs administration schedule in young C57BL/6 mice. (**B**) The body weight of mice. the spherical test results were not satisfied (*p* = 0.01), the difference between groups was significant (*p* = 0.03), and interaction is not existed (time*group = 0.08). The body weight at each time point was analyzed separately and the superscript means the difference degree between RmEVs and CmEVs/Blank. (**C**) The length of the whole intestine of mice. (**D**) The images of HE dye in the jejunum, scale bar: 200 μm. (**E**) The crypt depth of jejunum. (**F**) The villi length of jejunum. (**G, H**) EdU positive cells observation and assay in the jejunum of mice. Sections were stained with EdU and DAPI, scale bar: 100 μm. The quantitative analysis of EdU positive cells by Image J. (**I**) The positive CCND-1 protein in the jejunum, scale bars: 50 μm. (**J, K**) The relative mRNA expression of IGF-1R and PCNA in the jejunum. Continuous data of body weight were expressed as mean ± SD, others are expressed as means ± SEM. *p < 0.05, **p < 0.01; ns, not significant

To study the effects of RmEVs on intestinal development, a total of 24 mice were allocated into three groups and were given the same volume of CmEVs, RmEVs, and PBS daily for 20 d by gavage, respectively (Fig. 2A). RmEVs significantly increased body weight gain from day 5 of the experiment. The body weight of mice from RmEVs was higher than that from the CmEVs and PBS at the end of the trial (Fig. 2B). Besides, RmEVs could promote intestinal development, as evidenced by the longer total intestinal length, improved intestinal morphology, and increased jejunal crypt depth and villus length in the RmEVs group (Fig. 2C-F). To further investigate whether RmEVs affect intestinal cell proliferation and renewal, EdU and immunostaining were performed. The ratio of EdU-positive in the jejunum was remarkably increased after RmEVs treatment (Fig. 2G and H). The jejunal Cyclin D1 (CCND-1) protein was also upregulated in RmEVs (Fig. 2I). It indicated that RmEVs promote intestinal cell proliferation, and these results were also confirmed by RT-qPCR in the quantification of IGF-1R and PCNA expression (Fig. 2J and K).

**Fig. 3:**
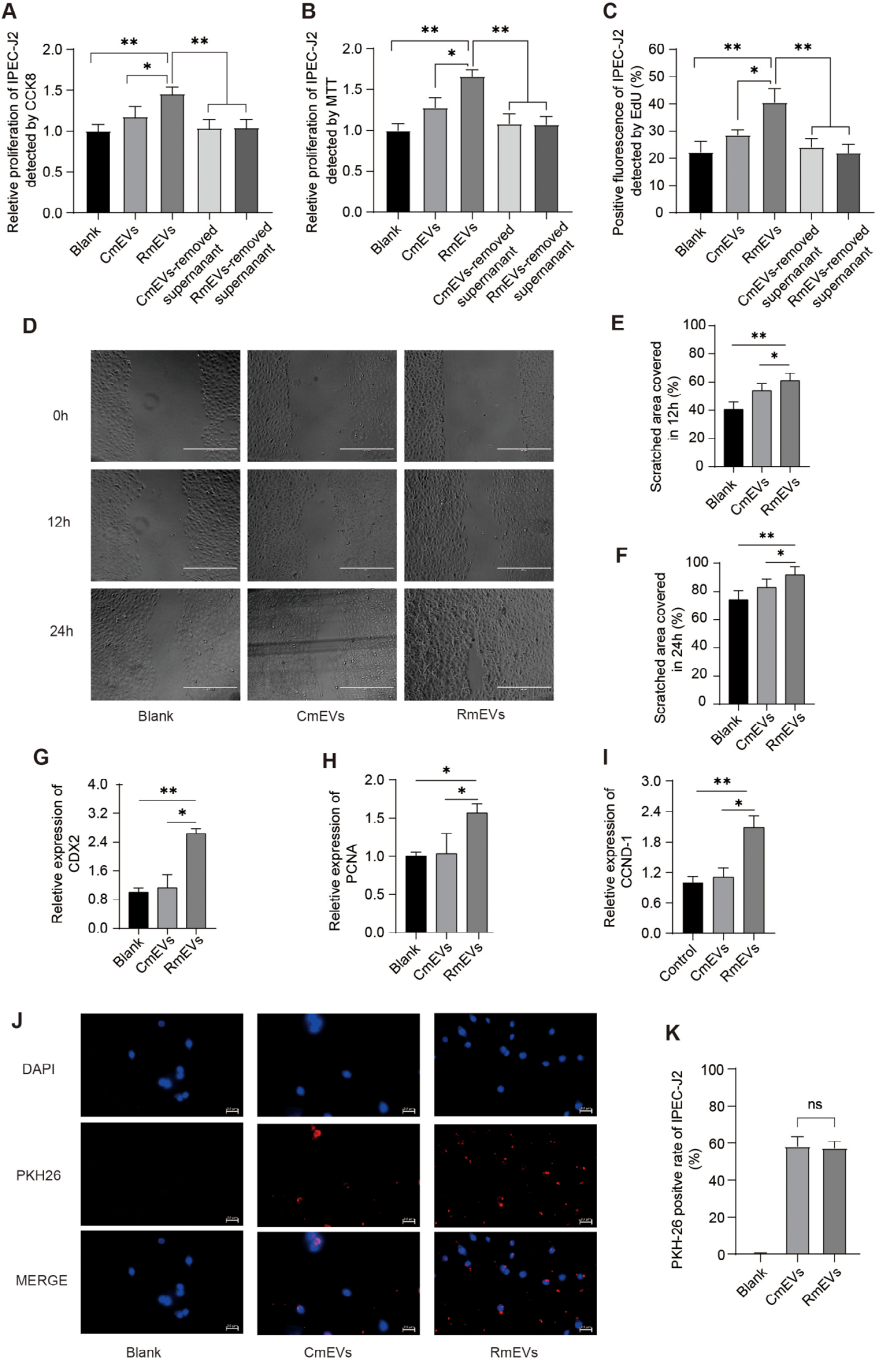
RmEVs promote cell proliferation and migration of IEPC-J2 (*n* = 3). (**A-C**) The assays of the proliferation of IPEC-J2 by CCK8 assay (**A**), MTT assay (**B**), and EdU assay (**C**). (**D-F**) Wound-healing assay. IPEC-J2 cells were treated with RmEVs, CmEVs, and Blank. Images were taken on the 0, 12, and 24 h after the scratch, scale bar: 400 μm. The spherical test results were not satisfied (*p* = 0.02), the difference between groups was significant (*p* = 0.04), and interaction did not exist (time*group = 0.22). The percentage of scratched area recovery of IPEC-J2 at 12 h **(E)** and 24 h (**F**) were analyzed separately. (**G-I**) The relative mRNA expression of CDX2, PCNA, and CCND-1. (**J**) Images of PKH26-labeled mEVs absorbed by IPEC-J2 after 6 h of culturing, scale bars = 50 μm. (**K**) The PKH-26 positive cell rates of IPEC-J2. Continuous data of recovery rate were expressed as mean ± SD, others are expressed as means ± SEM. *p < 0.05, **p < 0.01; ns, not significant

To understand the role of RmEVs on intestinal development, a porcine small intestinal epithelial cell line (IPEC-J2) cells model was used in vitro culture experiments. Firstly, we compared the effects of different concentrations of mEVs and treatment time on cell proliferation. As shown in Additional file 1: Figures S2A and S2B, the optimal treatment conditions of mEVs on IPEC-J2 was 200 µg/mL for 24 h. Next, to determine whether they directly mediate the beneficial effects of cell proliferation, IPEC-J2 cells were cultured with mEVs, or mEVs-removed supernatant from either milk of sows fed normal or RS-supplemented diets. As expected, both CmEVs and RmEVs could increase IPEC-J2 cell viability, while neither CmEVs-removed supernatant nor RmEVs-removed supernatant has any effects on cell viability (Fig. 3A and B). In consistency with the results of cell viability, the positive EdU fluorescence rate of RmEVs was remarkably higher than that of CmEVs, but there was no difference between CmEVs-removed supernatant and RmEVs-removed supernatant (Fig. 3C). In wound healing experiments, incubation with RmEVs increased by 10% and 18% wound healing rate compared to the incubation with CmEVs and PBS after 12 h, respectively (Fig. 3D and E), and the same trend was also found at 24 h after incubation with RmEVs, the wound healing rate increased by 9% and 17% than CmEVs and Blank (Fig. 3D and F). Similarly, the relative mRNA expression of PCNA, CDX2, and CCND-1 mRNA expression in the IPEC-J2 cells incubated with RmEVs were also strikingly higher than that expressed in CmEVs and Blank (Fig. 3G-I). In a mEVs absorption tracking experiment, CmEVs and RmEVs could be internalized and absorbed by IPEC-J2 cells (Fig. 3J). However, the PKH-26 positive cell rate in CmEVs was similar to that in RmEVs (Fig. 3K).

### The comparison of miRNA profiles and the identification of functional miRNAs that promote cell proliferation and migration

Given that the beneficial functions of RmEVs in promoting cell growth were not achieved by increasing the absorption efficiency into intestinal cells, we carried out miRNA sequencing to investigate whether miRNAs of RmEVs may contribute to the differentiation and viability of the intestinal epithelium. As shown in Table 1 and Additional file 1: Figure S3A, 7 miRNAs were upregulated and 2 miRNAs were downregulated in RmEVs. Predicted functional enrichment analysis of changed miRNAs indicated that MAPK signaling pathway, endocytosis, pantothenate, and CoA biosynthesis were at the top of the list (Additional file 1: Figure S3B).

**Table 1:** The expression and fold changes (FC) of miRNAs profiles between RmEVs and CmEVs

miRNAs	CmEVs ^a^	RmEVs ^b^	Log_2_FC (RmEVs/CmEVs)	p-value	t-value
miR-6516	43.36 ± 8.20	281.55 ± 55.18	2.708	0.006	-7.40
miR-1285	14356.88 ± 2131.81	39985.57 ± 10624.31	1.942	0.006	-4.09
miR-223	7220.70 ± 1906.24	21259.72 ± 5239.20	1.364	0.002	-4.36
miR-9841-3p	2.11 ± 1.19	55.28 ± 9.64	4.821	0.0002	-9.39
miR-18a	10.98 ± 3.73	291.0535 ± 65.46	4.738	0.002	-6.31
miR-146a-5p	31.42 ± 9.51	124.30 ± 29.91	1.959	0.004	-5.12
miR-142-5p	145.40 ± 24.92	316.79 ± 20.03	1.099	< 0.001	-9.28

^a^ CmEVs, milk-derived extracellular vesicles from Control; ^b^ RmEVs, milk-derived extracellular vesicles from maternal dietary RS group. Results were presented as mean ± SD

**Fig. 4:**
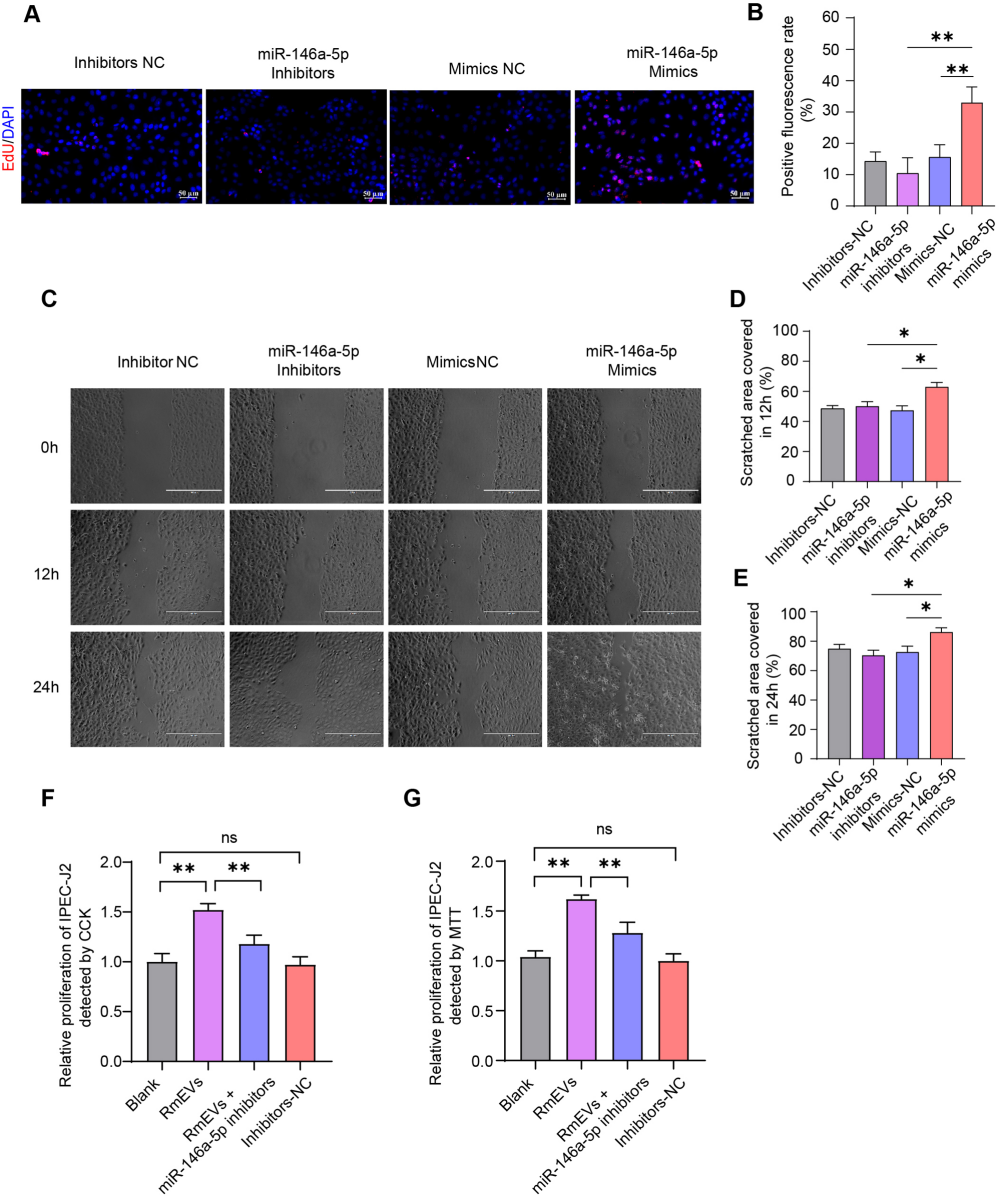
miR-146a-5p increases the proliferation and migration of IPEC-J2 (*n* = 3). (**A**) The images of EdU, scale bar: 50 μm. (**B**) The percentages of EdU in IPEC-J2. (**C**) Images of scratched recovery of IPEC-J2 cells after being transfected by miR-146a-5p mimics, inhibitors, mimics-NC, inhibitors-NC, scale bar: 400 μm. (**D, E**) The percentage of scratched area recovery of IPEC-J2. Data were analyzed by repeated measures analysis of variance, followed by within-group comparisons. The spherical test results were not satisfied (*p* = 0.04), the difference between groups was significant (*p* = 0.001), and interaction did not exist (time*group = 0.86). The scratched area recovery of IPEC-J2 at 12 h (**D**) and 24 h (**E**) were analyzed separately. (**F, G**) The cell proliferation of IPEC-J2 treated with RmEVs + inhibitors-NC, RmEVs + miR-146a-5p inhibitors, or Inhibitors-NC for 24 h followed by CCK (**F**) and MTT assays (**G**). Continuous data of recovery rate were expressed as mean ± SD, others are expressed as means ± SEM. *p < 0.05, **p < 0.01

Consistently, RT-qPCR confirmed that the levels of miR-6516, miR-1285, miR-223, miR-9841-3p, miR-18a, miR-146a-5p were significantly higher in RmEVs, and there was a higher trend for miR-142-5p in RmEVs. Besides, the 7 upregulated miRNAs showed a lower level in mEVs-deleted supernatant (≈ 1–2% of the miRNA level expressed in mEVs) (Additional file 1: Figure S3C-I). To investigate the effects of abundant miRNA in RmEVs on the proliferation of intestinal cells, the cell viability after miRNA mimics, inhibitors, or NC transfection were detected. As shown in Additional file 1: Figure S3J and S3K, miR-223, miR-146a-5p, and miR-18a significantly increased cell viability compared with their inhibitors and mimics-NC. Moreover, the miR-146a-5p showed a more efficient effect in enhancing cell viability than miR-223 and miR-18a, implying that the highly-expressed miR-146a-5p in RmEVs is helpful for the proliferation of IPEC-J2 cells. This result also be verified by the EdU assay, implying the fluorescence rate of IPEC-J2 transfected with miR-146a-5p mimics was higher than that of the mimics-NC and inhibitor (Fig. 4A and B). Furthermore, the wound healing rates were increased by 17% and 16% in miR-146a-5p mimics compared with inhibitors after 12 and 24 h, respectively (Fig. 4C-E). As shown in Fig. 4F and G, inhibition of miR-146a-5p remarkably weakened the promoting effect of RmEVs on the proliferation of IPEC-J2 cells. Taken together, these results suggest that miR-146a-5p is the key effector in RmEVs.

### The roles of miR-146a-5p in promoting cell proliferation are achieved by activating the β-catenin/Wnt pathway via targeting NEDD4L

**Fig. 5:**
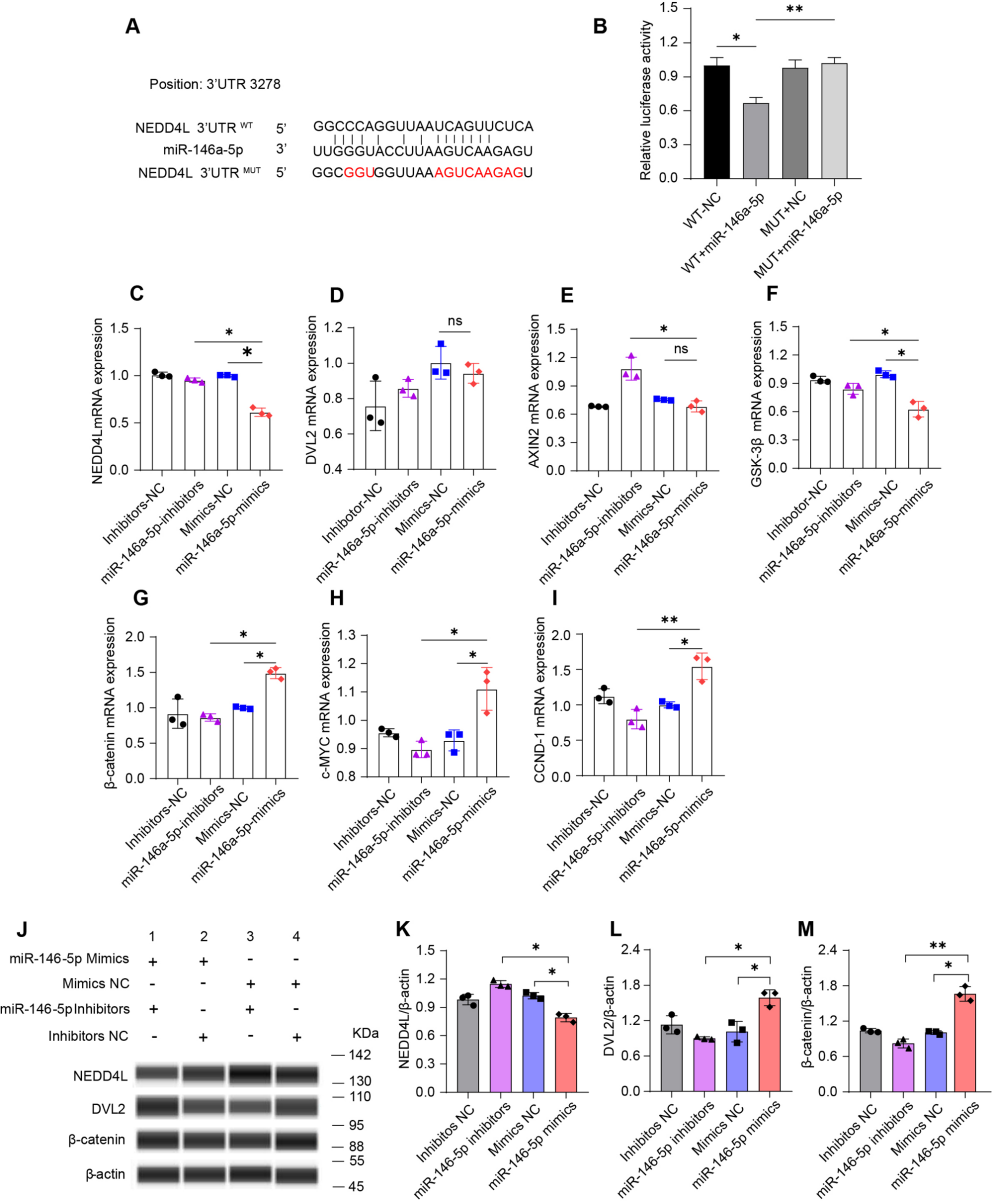
miR-146a-5p enhances the activation of Wnt pathway via targeting NEDD4L (*n* = 3). (**A**) The potential binding sites of miR-146a-5p in NEDD4L 3’UTR and their wild (WT) or mutant (MUT) sequence designs. (**B**) Luciferase reporter assays. HEK239 cells were co-transfected with pirGLO vector-WT/MUT and miR-146a-5p mimics or negative control. WT, wild type; MUT, mutant type; NC, negative control. (**C-I**) The relative mRNA expression of NEDD4L, DVL2, AXIN2, c-MYC, GSK-3β, β-catenin, and CCND-1 in IPEC-J2 cells after transfected with miR-146a-5p. (**J-M**) The protein levels of NEDD4L, DVL2, and β-catenin after transfected with miR-146a-5p. Data are expressed as means ± SEM. *p < 0.05, **p < 0.01; ns, not significant

Predictive analysis was performed by using Randa and Hybrid platforms. The neural precursor cell expressed developmentally downregulated 4-like (NEDD4L) was found to be the putative target gene of miR-146b-5p in both platforms. Moreover, we determined the alignment between miR-142-5p and 3′UTRs of NEDD4L sequences and found that position 3287 of 3′UTRs may be the potential binding site of miR-146a-5p (Fig. 5A). To verify this targeting, the wild-type (WT) and mutated-type (MUT) of putative binding sites were cloned into luciferase vectors. The constructed vectors were co-transfected into the HEK293. The luciferase intensity was significantly decreased by 32% when it was co-transfected with WT and miR-146-5p mimics, but not changed in the luciferase vector carrying a mutated-binding site (Fig. 5B). Moreover, the mRNA expression of NEDD4L was significantly decreased in IPEC-J2 when transfected with miR-146-5p mimics (Fig. 5C).

Previous studies indicated that the Wnt pathway may be the key for NEDD4L to participate in the regulation of early intestinal development [37, 38]. In our study, transfection of miR-146a-5p mimics failed to affect the mRNA expression of DVL2 and AXIN2, but decreased the mRNA expression of GSK-3β (Fig. 5D-F). However, the relative expression of β-catenin was increased after miR-146a-5p mimics transfection, indicating a positive regulation on the stability of β-catenin (Fig. 5G). As the target genes of Wnt, the expressions of c-MYC and CCDN-1 were also significantly up-regulated in the miR-146a-5p mimics group (Fig. 5H and I). The protein level of NEDD4L decreased by 30% in the miR-146a-5p transfection group (Fig. 5J and K; For high resolution, see Additional file 1: Figure S4A). Interestingly, overexpression of miR-146a-5p improved the protein level of DVL2 without affecting DVL2 mRNA expression (Fig. 5D and L). Besides, the protein level of β-catenin in mimics transfected IPEC-J2 cells was increased by 60% compared to mimics-NC (Fig. 5M).

### miR-146a-5p activates the β-catenin/Wnt pathway by inhibiting DVL2 proteasomal degradation

**Fig. 6:**
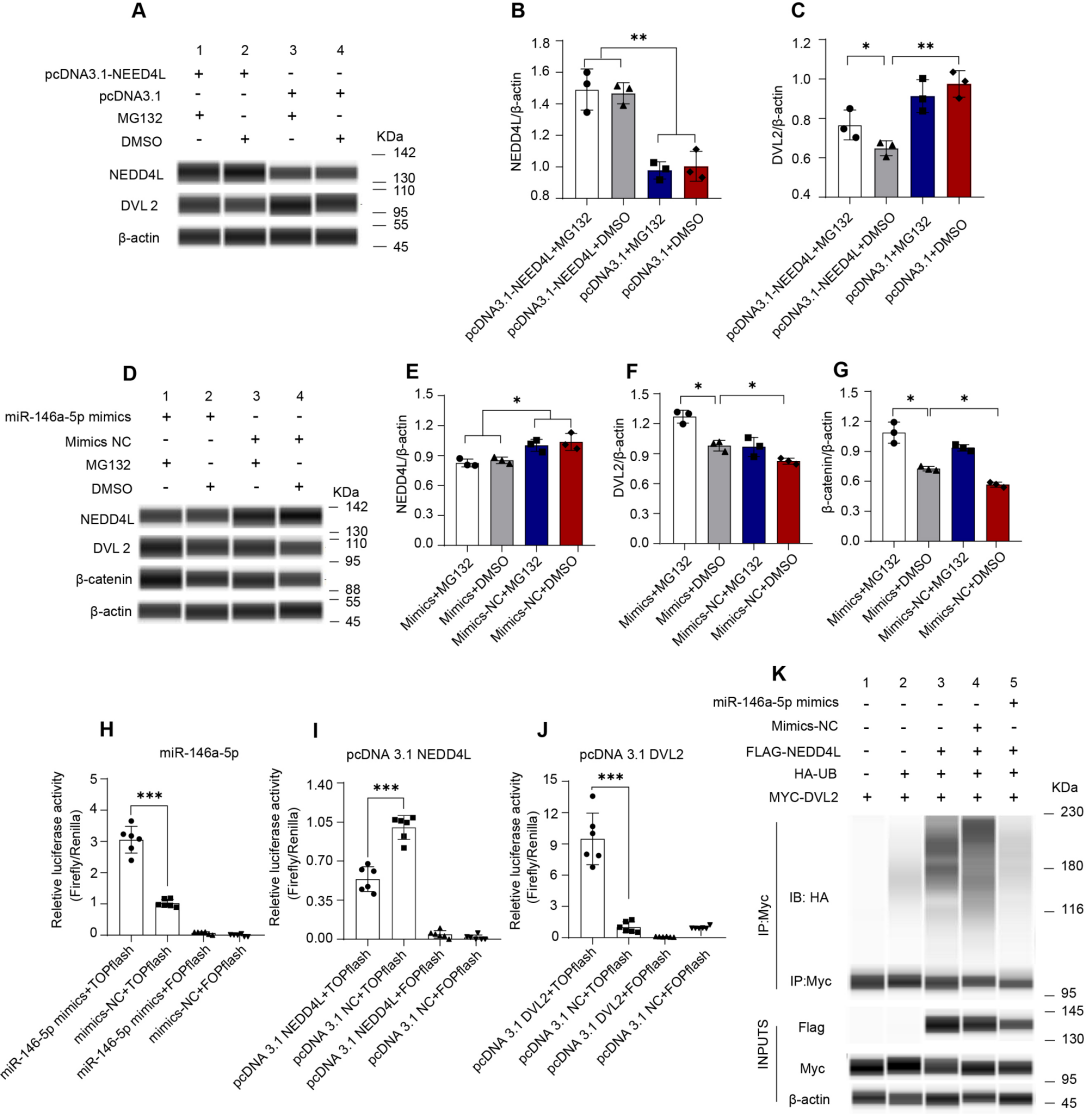
miR-146a-5p reduces proteasomal degradation of DVL2 via silencing the NEDD4L expression and modulates Wnt/β-catenin signaling (*n* = 3). (**A-C**) The protein expression levels of NEDD4L and DVL2 in IPEC-J2 cells were transfected with pcDNA3.1-NEDD4L or pcDNA3.1-control, then treated with MG132 or DMSO, and followed by Western blot analysis. (**D-G**) The protein expression levels of NEDD4L, DVL2, and β-catenin in IPEC-J2 cells were transfected with miR-146a-5p mimics or NC, then treated with MG132 or DMSO, and followed by Western blot analysis. (**H-J**) The relative TOP/FOP Flash reporter activities of IPEC-J2 cells after transfected with miR-146a-5p mimics or NC (**H**), pcDNA3.1-NEDD4L or pcDNA3.1-control (**I**), pcDNA3.1-DVL2 or pcDNA3.1-control (**J**). (**K**) Western blot images of HEK293 cells were transfected with constructs expressing Flag-NEDD4L, HA-Ubiquitin (HA-UB), MYC-DVL2, and miR-146-5p mimics or mimics-NC, then followed by anti-MYC IP under denaturing conditions. Data are expressed as means ± SEM. *p < 0.05, **p < 0.01, ***p < 0.001

To verify that NEDD4L is the target of miR-146a-5p to regulate DVL2 turnover, IPEC-J2 cells were overexpressed with pcDNA3.1-NEDD4L, followed by a western blotting analysis. The expression of NEDD4L was strongly induced by the transfection of pcDNA3.1-NEDD4L, which was not affected by proteasomal inhibitor MG132 (Fig. 6A and B; For high resolution, see Additional file 1: Figure S4B). The ubiquitination of DVL2 increased efficiently when co-transfected with the pcDNA3.1-NEDD4L, but it was reversed by the presence of MG132 (Fig. 6C), implying that NEDD4L targets DVL2 for proteasomal degradation via its E3 ligase activity. To further understand the role of miR-146a-5p in DVL2 proteasomal degradation, MG132 was added to overexpression systems. Transfection of miR-146a-5p decreased the level of NEDD4L protein without the influence of MG132 intervention (Fig. 6D and E; For high resolution, see Additional file 1: Figure S4C). Moreover, transfection of miR-146a-5p also can increase the levels of DVL2 and β-catenin protein, and these effects were enhanced by MG132 addition (Fig. 6D-F).

Next, to investigate the roles of miR-146a-5p, NEDD4L, and DVL2 in β-catenin-dependent transcription and Wnt pathway, IPEC-J2 were co-transfected with miR-146a-5p mimics or mimics NC, pcDNA3.1-NEDD4L and pcDNA3.1, or pcDNA3.1-DVL2 and pcDNA3.1, followed by transfection of a luciferase vector TOPflash or its negative control FOPflash (Fig. 6H-J). TOP Flash reporter activity increased in the cells in which miR-146a-5p was overexpressed, and FOP Flash activity was not affected when IPEC-J2 was transfected with miR-146a-5p (Fig. 6H). In addition, transfection of pcDNA3.1-NEDD4L resulted in reduced TOP activity, indicating expression of NEDD4L in the IPEC-J2 cell line strongly suppressed Wnt pathway activation (Fig. 6I). Overexpressing DVL2 in IPEC-J2 increased TOP reporter activity compared to controls (Fig. 6J), which means DVL2 participates in β-catenin secretion. Moreover, luciferase intensity could only be measured in TOP Flash but not in FOP Flash in all experiments. These results showed that miR-146a-5p and DVL2 are the positive regulators, while NEDD4L is the negative regulator in β-catenin/Wnt signaling.

To further confirm miR-146a-5p regulates DVL2 turnover through silencing the activity of the NEDD4L-mediated ubiquitination degradation, HEK293 were pre-treated with miR-146a-5p mimics or mimics-NC before overexpression of FLAG-NEDD4L, MYC-DVL2, and HA-Ubiquitin. As shown in Fig. 6K (For high resolution, see Additional file 1: Figure S4D), the ubiquitination of DVL2, indicated by the protein HA, was enhanced by the presence of FLAG-NEED4L. Moreover, DVL2 ubiquitination was reduced efficiently when co-expressed with the FLAG-NEDD4L and miR-146a-5p mimics, which may be due to the lower protein level of NEDD4L. DVL2 ubiquitination did not change in IPEC-J2 when co-transfected with mimics-NC and FLAG-NEDD4L.

### miR-146a-5p accelerates the early proliferation of intestinal cells in vivo

**Fig. 7:**
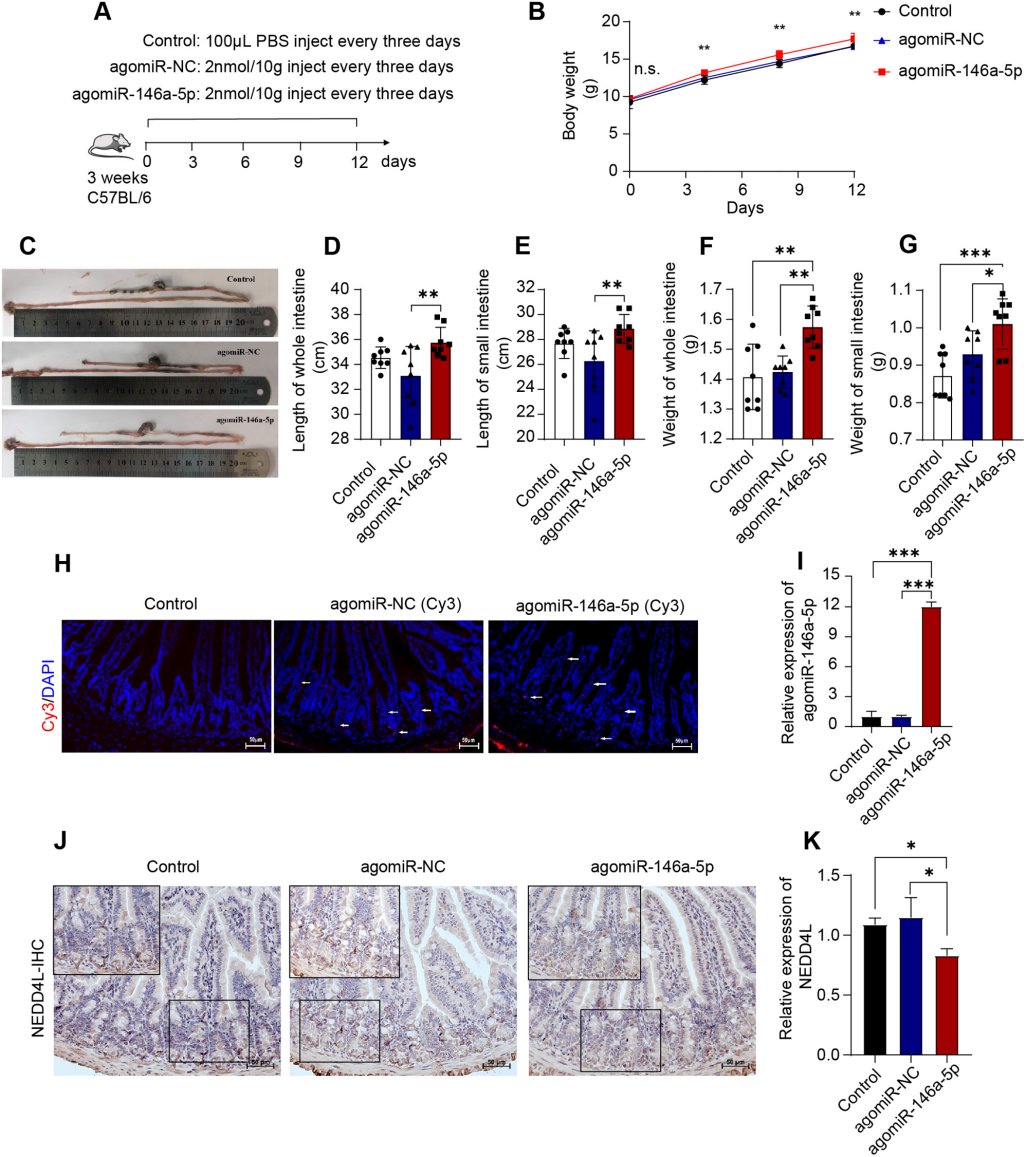
Overexpression of miR-146a-5p improves renewal of intestinal cells in young mice models (*n* = 8). (**A**) Animal designs and schedule. (**B**) The changes in body weight during the experiment. The spherical test results were satisfied (*p* = 0.144), the difference between groups was significant (*p* = 0.01), and interaction did not exist (time*group = 0.06). The body weight at each time point was analyzed separately and the superscript means the difference degree between agomiR-146a-5p and NC/Control. (**C**) The representative images of the intestine of mice from different treatments. (**D-G**) Quantitative analysis of intestinal length and intestinal weight of mice from different treatments. (**H**) Images of Cy3 and DAPI fluorescence in the jejunum of mice at 6 h after the injection of Cy3-labeled agomiR-NC, Cy3-labeled agomiR-146a-5p, or PBS. Scale bar: 50 μm. (**I**) The expression of miR-146a-5p in the jejunum. (**J, K**) Images of NEDD4L protein expression in the jejunum, presented by IHC method and quantitative analysis, scale bar: 50 μm. Continuous data of body weight were expressed as mean ± SD, others are expressed as means ± SEM. *p < 0.05, **p < 0.01, ***p < 0.001; ns, not significant

As shown in Fig. 7A, agomiR-146a-5p, agomiR-NC (2 nmol/10 g body weight, once every three days), or PBS (Control) was injected into the 21-days-old C57BL/6 weanling mice for 12 d. The body weight of mice in agomiR-146a-5p was higher than that in agomiR-NC and Control (Fig. 7B). The lengths and the weights of the whole intestine or small intestine were highest in agomiR-146a-5p (Fig. 7C-G). Besides, the jejunal morphologies (villi length and crypt depth) were also improved in agomiR-146a-5p injected mice (Additional file 1: Figure S4A and S4B). The results of EdU staining displayed that agomiR-146a-5p injection increases the proliferation rate of the jejunum cells (Additional file 1: Figure S4A and S4C).

In addition, the Cy3-labeled agomiR-146a-5p and Cy3-labeled agomiR-NC were injected into mice by intraperitoneal injection for 12 h before sacrifice (2nmol/10 g bodyweight). Confocal imaging of the jejunum section confirmed that both Cy3-labeled agomiR-146a-5p and Cy3-labeled agomiR-NC can be transported into intestinal epithelium from the abdominal (Fig. 7H). The results were also verified by the higher expression of miR-146a-5p in the jejunum in the agomiR-146a-5p group compared with the agomiR-NC and Control groups (Fig. 7I). IHC observation showed that the NEDD4L protein was scattered throughout the intestinal crypts and villi. The positive rate of NEDD4L protein decreased in the jejunum of agomiR-146a-5p mice (Fig. 7J). Consistently, the relative gene level of NEDD4L was significantly downregulated in agomiR-146a-5p, but not changed in agomiR-NC (Fig. 7K). Moreover, the positive number of β-catenin and CCND-1 protein increased in the jejunum of agomiR-146a-5p injected mice (Additional file 1: Figure S5A and S5D-F), suggesting that the enhanced cell renewal capacity was accompanied by the activation of Wnt signaling. Besides, a significant reduction in cleaved-caspase3 protein was also found in agomiR-146a-5p (Additional file 1: Figure S5A and S5G). Taken together, our data indicated that miR-146a-5p decreases the NEDD4L gene expression, resulting in the activation of the Wnt pathway and thereby improving intestinal proliferation and renewal.

## Discussion

The mEVs in breast milk are essential for the intestinal development of offspring. Maternal diet structure can influence miRNAs expression in mEVs, thereby regulating the health of infants. In the present study, we proposed that mEVs from the mother who consumes fiber-rich can promote intestinal development in offspring. We discovered the health-promoting effects of mEVs are achieved through the enrichment of miR-146a-5p. Moreover, maternal fiber-rich miR-146a-5p can target the 3’UTR of ubiquitin ligase gene NEDD4L in the ubiquitin-proteasome system, thereby reducing the expression of NEDD4L. The reduction of the early ubiquitination process contributes to the reduction of DVL2 proteasomal degradation that enhances the Wnt activation, thus promoting the renewal of intestinal cells in offspring.

A high-quality maternal diet is beneficial to the early development of infants. Consumption of inadequate diet during late gestation and lactation may increase the metabolic risk in offspring [39]. Noteworthily, maternal dietary fiber can mitigate the negative effects of the maternal imbalance diet on offspring [40–42]. Moreover, a maternal fiber diet markedly increases the depth of the ileal crypt and the levels of intestinal proteins involved in antioxidant abilities, energy metabolism, and immune responses, and decreases the level of proteins related to apoptosis and cell motility [43]. In this study, the maternal RS diet promoted intestinal development, weanling health, and the concentration of serum growth factor in offspring. Breastfeeding is an effective way for offspring to receive maternal signal transduction including immune regulation and nutrient metabolism [44]. Some people found maternal RS diet intake reduces the protein concentration in milk, and it still controversial [32]. In the present study, we did not find any changes in milk quality after maternal RS intake, nor did we see significant concentrations of some immunoglobulins. Therefore, small molecular particles in breast milk may be the key to this research. Previous studies have revealed mEVs carrying a wide range of functional proteins, mRNA, and miRNA, that mediate intercellular communications [8] and promote gut development and health [45, 46]. Therefore, we focused on the role of mEVs in maternal diet affecting offspring development and health. Growing mouses aged 3 to 6 weeks were chosen to investigate intestinal development in this study because of the good growth performance and intestinal cell renewal rate of mouses during this period [47, 48]. As we observed, mEVs, especially the mEVs after maternal RS supplementation, can be absorbed by intestinal cells, and then promote intestinal cell proliferation and development both in vivo and in vitro. The physical properties of mEVs were not significantly changed after maternal RS supplementation, it was most likely due to the alterations of the active ingredients in mEVs, these effects were also verified by other studies [24, 25].

MiRNAs are involved in gene silencing and degradation and play a key regulatory role in various biological development processes [49], including cell differentiation, proliferation, development, and homeostasis [50]. MiRNAs could be absorbed by the intestinal epithelium along with mEVs into the target cells. Even, the lipid membrane of mEVs helps to protect mEVs-encapsulated miRNAs against degradation by ribonucleases, low pH, and digestive enzymes [9, 51], and thereby protect the important functions of mEVs-encapsulated miRNAs in the communication from mother to child [8]. Hundreds of miRNAs have been identified in human and mammalian mEVs, some of which were predicted to be associated with cellular signaling, immunity, and cell proliferation [52–54]. We uncovered that the miR-146a-5p, enriched in maternal fiber-rich mEVs, is associated with intestinal cell proliferation in early growth. A previous study suggested that miR-146a-5p can inhibit inflammation and the occurrence of autophagy [55]. Besides, miR-146a-5p increases the regeneration of intestinal epithelial cells and protects LPS-induced injury by targeting the NF-κB [56]. Exosomal miRNA-146a-5p from the amniotic fluid-derived stem cells promoted skin regeneration [57]. Consistent with previous research, our study confirmed that miR-146a-5p encapsulated by mEVs promotes intestinal development in young animals.

The Wnt signaling pathway is composed of a large family of secreted proteins that can induce evolutionarily conserved intracellular signals and influence different cellular responses during development [58]. The Wnt pathway is also associated with embryonic development and early neonatal development [59]. For example, Wnt is involved in the development of intestinal cells, and its downstream proteins regulate the cell cycle and regeneration [60]. When the Wnt protein is activated, β-catenin accumulates in the cell and is then translocated to TCF/β-catenin transcriptional activator in the nucleus, which eventually initiates the transcription of the Wnt and specific downstream target genes [59]. However, the cytoplasmic pool of β-catenin typically is degraded by a complex protein scaffold consisting of adenomatous polyposis coli protein, β-catenin, and GSK-3β kinase, thus keeping β-catenin at a low level [60]. DVL2 can inhibit the production of this protein complex, thereby promoting the normal operation of the β-catenin/Wnt signaling [61]. Moreover, mediating DVL2 degradation has become the main pathway to regulate the activity of the Wnt pathway in cell [62]. Recently, miRNAs interfering is an effective way to regulate the Wnt pathway. For example, intestinal miR-802 regulates enterocyte differentiation and epithelial proliferation by depressing the Tmed9, a modulator of Wnt [63]. In our study, miR-146a-5p promotes DVL2 protein levels by inhibiting the ubiquitination degradation of DVL2. Besides, both overexpression and TOP/FOP flash assay verified that miR-146a-5p promoted the proliferation of intestinal cells by activating the Wnt pathway.

Ubiquitination is a common protein modification that induces the stabilization, degradation, or reorientation of protein substrates, thereby affecting the activity of metabolic pathways [64]. Many proteins are degraded by proteasomes or lysosomes after ubiquitination, including cell cycle regulators, membrane proteins, tumor suppressors, and transcription factors [65, 66]. Meanwhile, NEDD4L is a ubiquitin-protein ligase in the NEDD4 family, which can bind and regulate membrane proteins and promote the internalization and turnover of membrane proteins [67]. The WW domains structure of NEDD4L have a high affinity for the PPxY (PY) motifs of protein, which mediating its specific binding to the substrates contain PY motifs [67]. Previous study showed that NEDD4L presents a higher concentration in the intestine, especially in the crept than in the villi [37]. Consistently, NEDD4L was found uniformly distributed laterally in the jejunum of mice in this study. NEDD4L-mediated protein degradation may affect the Wnt pathway and the cell cycle and thus affect overall tissue change. Moreover, NEDD4L potentially inhibit Wnt signaling by targeting DVL protein for proteasomal degradation, as DVL2 protein contains two PY motifs in the C-terminal region [68, 69]. In this study, we found that the overexpression of NEDD4L degraded the amount of DVL2 protein, thereby inhibiting the activation of the Wnt pathway, while overexpression of miR-146a-5p counteracts these effects. Moreover, a miRNA-knockout mice model will be built in our future work to investigate the effects of miR-146a-5p knockout on the quality of maternally-produced mEVs and offspring health.

## Conclusion

In conclusion, the present study demonstrated that maternal RS consumption promotes the growth and intestinal health of offspring during early life. The mEVs is an important effector in maternal RS supplement influence the intestinal development of offspring. Furthermore, miR-146a-5p was identified to be enriched in mEVs after maternal RS diet intake, which can target to silence NEDD4L gene expression, and subsequently reduce the degradation of DVL2, thereby activating the Wnt pathway in the intestine. These findings highlight the importance of mEVs in the connection between maternal diet and infant health, which suggests a strategy for how to regulate mEVs by regulating maternal diet, thereby to promote early intestinal development in offspring.

### Electronic supplementary material

Below is the link to the electronic supplementary material.

**Supplementary Material 1: Table S1.** Ingredients and nutrient compositions of experimental diets. **Table S2. **The sequences of primer for the RT-qPCR measurement of miRNAs and mRNAs. **Table S3. **The antibodies, reagents, commercial assays, software, and other resource used in this study. **Table S4. **The effects of maternal dietary RS intervention on growth performance of suckling piglets. **Table S5.** The effects of maternal dietary Resistant starch intervention during lactation on milk quality of sows. **Figure S1.** Characterizations of mEVs and mEVs-removed supernatant isolated from sow milk. (A) Physical characteristics observed by TEM, scale bars = 200 nm. (B) The size distribution of mEVs is measured by Nanosight. (C) The marker protein expression in mEVs and mEVs-removed supernatant. (D) The concentration of particles of mEVs and mEVs-removed supernatant. **Figure S2.** Gradient experimental design to explore the optimal treatment concentration and treatment time of mEVs in IPEC-J2 cell line. (A) The gradient experimental design for optimal treatment time. The spherical test results were not satisfied (*p* = 0.01), the difference between groups was significant (*p* = 0.03), and interaction did not exist (time*group = 0.15). The OD of cell culture at each time point was analyzed separately and data are expressed as means ± SD. (B) The gradient experimental design for optimal concentration, data are expressed as means ± SEM. Differences in superscript letters for the peer data indicate that the difference is significant (*p* < 0.05). **Figure S3.** The miRNA expression profiles in two kinds of mEVs and their effects on the proliferation of IPEC-J2 cells. (A) The volcanic map of expression of miRNAs between the CmEVs and RmEVs. Red, upregulated in RmEVs. Blue, upregulated in CmEVs. (B) KEGG pathway analysis of target genes of miRNAs differently expressed in CmEVs and RmEVs. (C-I) The relative expression of miR-6516, miR-223, miR-1285, miR-9841-3p, miR-18a, miR-146a-5p, miR-142-5p in CmEVs, RmEVs, and their supernatants measured by RT-qPCR. (J, K) The cell proliferation of IPEC-J2 after transfected by mimics, inhibitors, and NC of miR-6516, miR-223, miR-1285, miR-9841-3p, miR-18a, miR-146a-5p, and miR-142-5p, respectively, then measured by MTT assay. Data are expressed as means ± SEM. *p < 0.05, **p < 0.01. **Figure S4.** The high-resolution images of Western blot obtained by the WES^TM^ System. (A) IPEC-J2 cells transfected with miR-146a-5p mimics. (B) IPEC-J2 cells were transfected with pcDNA3.1-NEDD4L or pcDNA3.1-control, then treated with MG132 or DMSO. (C) IPEC-J2 cells were transfected with miR-146a-5p mimics or NC, then treated with MG132 or DMSO. (D) HEK293 cells were transfected with constructs expressing Flag-NEDD4L, HA-Ubiquitin (HA-UB), MYC-DVL2, and miR-146-5p mimics or mimics-NC, followed by anti-MYC IP detection. **Figure S5. **Overexpression of miR-146a-5p increases the proliferation and renewal of intestinal cells in mice model (n = 8). (A) Representative images of jejunum stained for HE, EdU, β-catenin, CCND1, and cleaved-caspase 3. Scale bars: 50 µm. (B, C) The villi length and crypt depth of jejunum. (D-G) The ratio of positive cells in jejunum for EdU, β-catenin, CCND1, and cleaved-caspase3. Data are expressed as means ± SEM. *p < 0.05, **p < 0.01

## Data Availability

Data is available only with the permission from corresponding author.
