# Supplementary material for: Maternal fiber-rich diet promotes early-life intestinal development in offspring through milk-derived extracellular vesicles carrying miR-146a-5p
Source: J Nanobiotechnology. 2024 Feb 16;22:65. doi: 10.1186/s12951-024-02344-4 (PMC10870446; doi:10.1186/s12951-024-02344-4)
Supplement: Supplementary file 1 — Supplementary Material 1: Table S1. Ingredients and nutrient compositions of experimental diets. Table S2. The sequences of primer for the RT-qPCR measurement of miRNAs and mRNAs. Table S3. The antibodies, reagents, commercial assays, software, and other resource used in this study. Table S4. The effects of maternal dietary RS intervention on growth performance of suckling piglets. Table S5. The effects of maternal dietary Resistant starch intervention during lactation on milk quality of sows. Figure S1. Characterizations of mEVs and mEVs-removed supernatant isolated from sow milk. (A) Physical characteristics observed by TEM, scale bars = 200 nm. (B) The size distribution of mEVs is measured by Nanosight. (C) The marker protein expression in mEVs and mEVs-removed supernatant. (D) The concentration of particles of mEVs and mEVs-removed supernatant. Figure S2. Gradient experimental design to explore the optimal treatment concentration and treatment time of mEVs in IPEC-J2 cell line. (A) The gradient experimental design for optimal treatment time. The spherical test results were not satisfied (p = 0.01), the difference between groups was significant (p = 0.03), and interaction did not exist (time*group = 0.15). The OD of cell culture at each time point was analyzed separately and data are expressed as means ± SD. (B) The gradient experimental design for optimal concentration, data are expressed as means ± SEM. Differences in superscript letters for the peer data indicate that the difference is significant (p < 0.05). Figure S3. The miRNA expression profiles in two kinds of mEVs and their effects on the proliferation of IPEC-J2 cells. (A) The volcanic map of expression of miRNAs between the CmEVs and RmEVs. Red, upregulated in RmEVs. Blue, upregulated in CmEVs. (B) KEGG pathway analysis of target genes of miRNAs differently expressed in CmEVs and RmEVs. (C-I) The relative expression of miR-6516, miR-223, miR-1285, miR-9841-3p, miR-18a, miR-146a-5p, miR-142-5p in CmEVs, [file 12951_2024_2344_MOESM1_ESM.docx]

Additional Information

**Table S1.** Ingredients and nutrient composition of experimental diets.

| Items | Control (CN) | Resistant starch (RS) |
| --- | --- | --- |
| Ingredient (%) |  |  |
| Corn | 53.50 | 53.50 |
| Soybean meal | 13.00 | 13.00 |
| Soybean oil | 2.00 | 2.00 |
| Wheat bran | 28.00 | 26.00 |
| Resistant starch | - | 2.00 |
| Limestone | 1.25 | 1.25 |
| CaHPO_4_ | 1.10 | 1.10 |
| NaCl | 0.50 | 0.50 |
| L-Lysine HCl | 0.05 | 0.05 |
| Methionine | 0.04 | 0.04 |
| Threonine | 0.05 | 0.05 |
| Tryptophan | 0.01 | 0.01 |
| Premix | 0.50 | 0.50 |
| Total | 100.00 | 100.00 |
| Nutrient composition ^a^ |  |  |
| DE (Mcal/kg) | 3.29 | 3.24 |
| CP (%) | 15.50 | 15.29 |
| Ca (%) | 0.78 | 0.78 |
| P (%) | 0.75 | 0.73 |
| Lys (%) | 0.73 | 0.72 |
| Met (%) | 0.30 | 0.29 |
| Thr (%) | 0.58 | 0.57 |
| Trp (%) | 0.17 | 0.17 |
| NDF (%) | 15.64 | 16.12 |
| ADF (%) | 4.92 | 4.77 |
| TDF (%) | 17.89 | 18.69 |

^a^ DE: Digestion energy, CP: Crude protein, Ca: Calcium, P: Phosphorus, Lys: lysine, Met: Methionine, Cys: Cysteine, Thr: Threonine, Trp: Tryptophan, NDF: Neutral detergent fiber, ADF: Acid Detergent Fiber, TDF: Total dietary fiber.

**Table S2.** The sequences of primer for the RT-qPCR measurement of miRNAs and mRNAs.

| miRNAs/mRNAs | Forward Primer | Reverse Primer |
| --- | --- | --- |
| miR-1285 | CTGGGCAACATAGCGAGACCCCGT | TGGTGTCGTGGAGTCG |
| miR-223 | TGTCAGTTTGTCAAATACCCC | TGGTGTCGTGGAGTCG |
| miR-6516 | ATCATGTATGATACTGCAAACA | TGGTGTCGTGGAGTCG |
| miR-146a-5p | TGAGAACTGAATTCCATGGGTT | TGGTGTCGTGGAGTCG |
| miR-9841-3p | TCTAGCATCGAGCACCCGCCT | TGGTGTCGTGGAGTCG |
| miR-18a | TAAGGTGCATCTAGTGCAGATA | TGGTGTCGTGGAGTCG |
| miR-142-5p | TTTTGATTAATATAGTGAAATTTCGT | TGGTGTCGTGGAGTCG |
| U6 | GGAACGATACAGAGAAGATTAGC | TGGAACGCTTCACGAATTTGCG |
| PCNA | GAAGAAGGTGCTGGAGGCT | TGGACATGCTGGTGAGGTT |
| CCND-1 | TGAGGAGCAGAAGTGCGAAGA | CGGCAGTCAAGGGAATGGT |
| CDX2 | ACCGCAGAGCCAAGGAGA | AGGAGGTCACAGGAGTCAAGG |
| IGF-1R | GGCAAGTATGCGTGAAAGAATC | CTAAAGGTCGGAGGAATGAGG |
| P53 | CATTGTCAGGCTTATGGAAACTAC | ACACTCGGAGGGCTTCACTT |
| NEDD4L | GCCCGTTCGTCAACTGTCA | GCGTCCCTTTGCATCCTTT |
| DVL2 | CAAGATCACCATCCCAAACG | CCGCCTTCAACAGCCCACT |
| AXIN2 | CTGAGGGCTACTGCGAATG | TCCATCTACGCTGCTGTCC |
| β-catenin | GACCATGCCATGATTGGACCTGAG | GCCTGTCAACCTTCTCGCTGTC |
| c-MYC | CTGCCAAGAGGGCTAAGTT | TCTGGCGTTCCAAGACATT |
| GSK-3β | CAGAGACAAGGATGGCAGCAAGG | TGGCAACCAGTTCTCCTGAATCAC |
| β-actin | TCGTGCGTGACATCAAAGA | AAGAAGGAAGGCTGGAAAA |

**Table S3.** The antibodies, reagents, commercial assays, softwares and other resource used in this study.

| Reagent or resource | Source | Identifier |
| --- | --- | --- |
| Antibodies | | |
| DVL2 | Proteintech | RRID:AB_2093330 |
| NEDD4L | Proteintech | RRID:AB_2149326 |
| β-catenin | Proteintech | RRID:AB_2086128 |
| Flag | Proteintech | RRID:AB_2918475 |
| HA | Proteintech | RRID:AB_2881490 |
| MYC | Proteintech | RRID:AB_11182162 |
| IgG | Proteintech | RRID:AB_2819035 |
| β-actin | Proteintech | RRID:AB_2923704 |
| CD63 | Proteintech | RRID:AB_2783831 |
| CD9 | Proteintech | RRID:AB_2878706 |
| Calnexin | Proteintech | RRID:AB_2069033 |
| CCND-1 | Servicebio | CAT#GB111372 |
| Cleaved-caspase3 | Servicebio | CAT#GB11532 |
| Chemicals, peptides, and recombinant proteins | | |
| PKH26 Red Fluorescent Cell Linker Kit | Bestbio | CAT#bb-441125 |
| DAPI | Solarbio | CAT#D8200 |
| Cell Counting Kit-8 (CCK8) | MedChemExpress | CAT#HY-K0301 |
| Thiazolyl Blue (MTT) | MedChemExpress | CAT#HY-15924 |
| RIPA Lysis and Extraction Buffer | Thermo Fisher Scientific | CAT#89900 |
| Protease Inhibitor Cocktail (EDTA-Free) | MedChemExpress | CAT#HY-K0010 |
| Fetal Bovine Serum | Gibico | CAT#10270-106 |
| DMEM high glucose | Servicebio | CAT#G4511 |
| DAPI | Solarbio | CAT#C0060 |
| Phosphate Buffered Saline | Servicebio | CAT#G0002 |
| MG132 | MedChemExpress | CAT#HY-13259 |
| 10% formalin | Servicebio | CAT#G1101 |
| Lipofectamine 2000 | Invitrogen | CAT#11668019 |
| Critical commercial assays | | |
| BCA Protein Assay Kit | Thermo Fisher Scientific | CAT#A53227 |
| RNApure extraction kit | Aidlad | CAT#RN0302 |
| Co-IP kit | Proteintech | CAT#PK10008 |
| Wes Separation module | ProteinSimple | SM-W004 |
| Dual-Luciferase Reporter Assay | Promega | CAT#E1910 |
| Mir-X miRNA First-Strand Synthesis Kit | Takara | CAT#638313 |
| SYBR Premix Ex Taq TM II | Takara | CAT#DRR081A |
| TruSeq Small RNA Sample Preparation Kit | Illumina | CAT#RS200-0012 |
| Experimental models: Cell lines | | |
| IPEC-J2 | DSMZ | RRID: CVCL_2246 |
| HEK293T | DSMZ | RRID: CVCL_0063 |
| Experimental models: Organisms/strains | | |
| Mouse: C57BL/6J | Sipeifu, Beijing | RRID: IMSR_JAX:000664 |
| Sows: Yorkshire × Landrace, ages ranged from 3-6 parity | Jiuyun animal husbandry, Chengde | N/A |
| Oligonucleotides | | |
| qRT-PCR primers | Sangon,Shanghai | See Table S2 |
| miRNA mimics/inhibitors | Sangon,Shanghai | N/A |
| agomiRNA and Cy3-labeled agomiRNA | Sangon,Shanghai | N/A |
| Recombinant DNA | | |
| pcDNA3.1-Flag-C | Qingke Biotech, Beijing | N/A |
| pCMV-Myc | Qingke Biotech, Beijing | N/A |
| pCMV-HA-C | Qingke Biotech, Beijing | N/A |
| pmirGLO | Promega | CAT#E1330 |
| TOPFlash/ FOPFlash | Beyotime | CAT#D2501 |
| pRL-TK/Renilla | Beyotime | CAT#D2760 |
| Software and algorithms |  |  |
| IBM SPSS Statistics 22 | IBM | RRID:SCR_016479 |
| Image J | National Institute of Health | RRID: SCR_003070 |
| Roche Light Cycler 480 qPCR Real Time PCR System | Roche | RRID:SCR_018626 |
| Compass for Simple Western | ProteinSimple | RRID:SCR_022930 |
| GraphPad Prism | GraphPad | RRID: SCR_002798 |
| SnapGene | SnapGene | RRID:SCR_015052 |
| Primer Designer 5 | PrimerDesigner | RRID:SCR_003189 |

**Table S4.** The effects of maternal dietary resistant starch intervention during lactation on growth performance of suckling piglets **(n = 20)**.

| Items | Control (CN) | Resistant starch (RS) | Between-group p-value | | t-value |
| --- | --- | --- | --- | --- | --- |
| Litter size, No./Litter | 12.57± 0.53 | 12.13 ± 0.55 | 0.25 | | 0.88 |
| Litter weight, kg |  | | |  |  |
| After cross-fostering | 17.86 ± 1.62 | 17.06 ± 1.25 | 0.83 | | 0.22 |
| At day 7 | 34.60 ± 3.12 | 33.90 ± 3.65 | 0.81 | | -0.24 |
| At day 14 | 53.68 ± 4.40 ^b^ | 57.21 ± 4.52^a^ | 0.04 | | -2.14 |
| At day 21 | 72.49 ± 5.86^b^ | 81.86 ± 6.91 ^a^ | 0.02 | | -2.42 |
| Within-group p-value | 0.00 | 0.00 | - | | - |
| Piglets mean weight, kg |  | | |  |  |
| After cross-fostering | 1.42 ± 0.20 | 1.47±0.18 | 0.41 | | -0.84 |
| At day 7 | 2.81 ± 0.22 | 2.96 ± 0.25 | 0.38 | | -0.88 |
| At day 14 | 4.40 ± 0.37^b^ | 4.93 ± 0.30^a^ | 0.02 | | -2.47 |
| At day 21 | 6.37 ± 0.52^b^ | 7.08 ± 0.45^a^ | 0.02 | | -3.15 |
| Within-group p-value | 0.00 | 0.00 | - | | - |
| Average daily weight gain (ADG), g/d |  | | |  |  |
| 1st week of lactation | 198.17 ± 39.24 | 207.65 ± 33.53 | 0.75 | | -0.32 |
| 2nd week of lactation | 233.45 ± 20.55^b^ | 287.55 ± 26.69^a^ | 0.02 | | -2.41 |
| 3rd week of lactation | 230.39 ± 28.73^b^ | 315.00 ± 35.92^a^ | 0.01 | | -3.29 |
| Within-group value | 0.00 | 0.00 | - | | - |
| Day 1-21 | 292.77 ± 20.95^b^ | 335.78 ± 25.37^a^ | 0.02 | | -2.72 |

Results are expressed as means ± SD.

For Litter weight, the spherical test results were not satisfied (p = 0.00). There are different trends among the groups (p = 0.08) and non-significant interaction (time*group = 0.06).

For the mean weight of piglets, the spherical test results were satisfied (p = 0.55), the difference between groups was significant (p = 0.02) and interaction existed (time*group = 0.04).

For Average daily weight gain, the spherical test results were not satisfied (p = 0.04). There are different trend among the groups (p= 0.00) and non-significant interaction (time*group = 0.165)

^a, b^ Mean values within a row with different letters differ significantly at p < 0.05.

**Table S5.** The effects of maternal dietary Resistant starch intervention during lactation on milk quality of sows **(n = 20)**.

| Items | Control (CN) | | Resistant starch (RS) | | p-value | t-value |
| --- | --- | --- | --- | --- | --- | --- |
| Colostrum, day 1 |  |  | |  |  |  |
| Fat (%) | 6.56 ± 0.77 | | 6.32 ± 0.81 | | 0.75 | 0.59 |
| Protein (%) | 15.58 ± 2.18 | | 15.84 ± 1.59 | | 0.34 | -0.87 |
| Lactose (%) | 1.90 ± 0.31 | | 1.85 ± 0.52 | | 0.55 | 0.77 |
| Total solids (%) | 23.04 ± 1.49 | | 23.37 ± 1.53 | | 0.66 | 0.69 |
| Milk, day 21 |  |  | |  |  |  |
| Fat (%) | 4.90 ± 0.48 | | 5.06 ± 0.41 | | 0.68 | -0.66 |
| Protein (%) | 5.38 ± 0.47 | | 5.28 ± 0.54 | | 0.44 | 0.80 |
| Lactose (%) | 6.02 ± 0.13 | | 6.18 ± 0.18 | | 0.29 | -0.93 |
| Total solids (%) | 16.90 ± 1.04 | | 17.11 ± 1.12 | | 0.89 | -0.49 |

Results were presented as means ± SD.


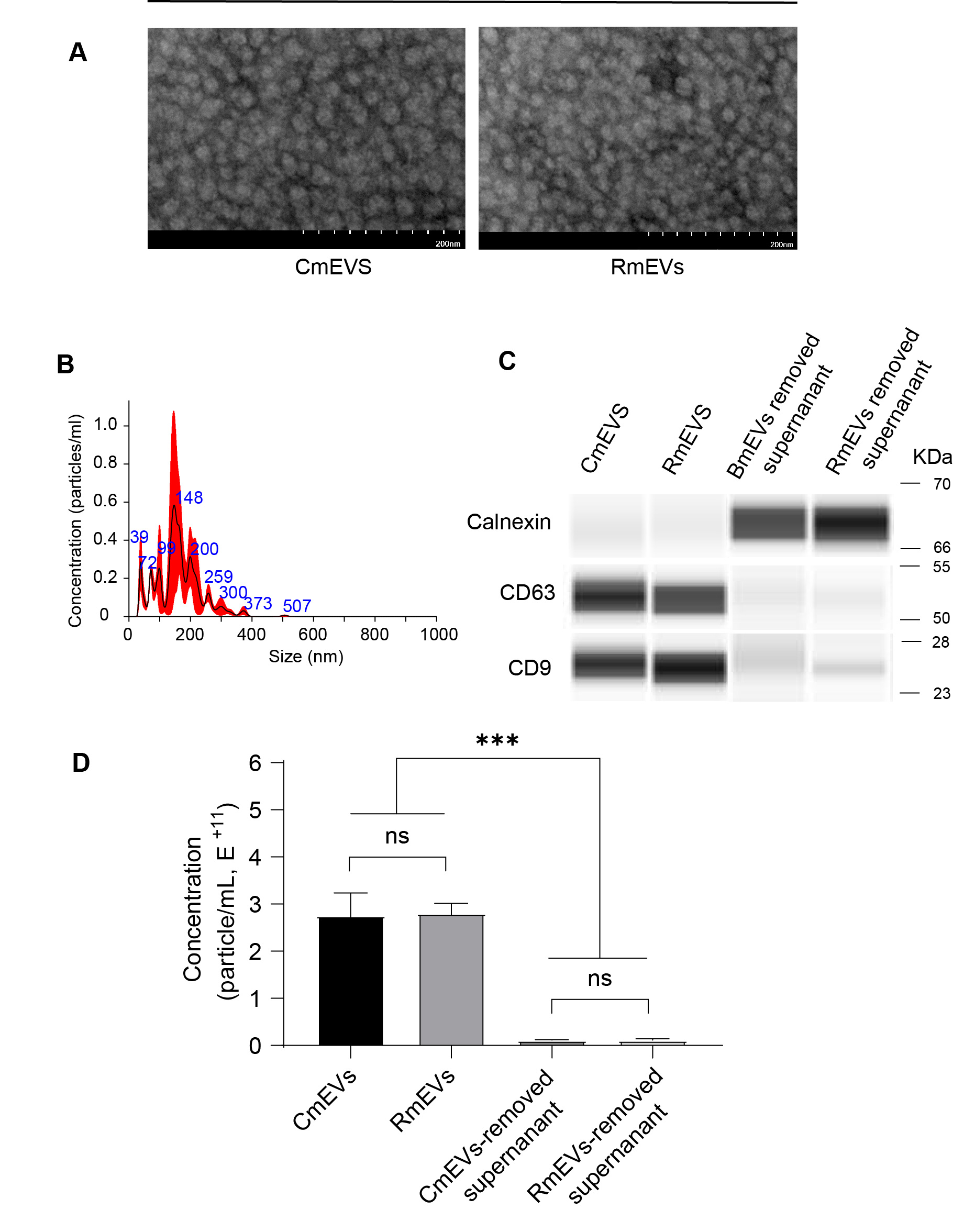


**Figure S1.** **Characterizations of milk-derived extracellular vesicles (mEVs) and mEVs-removed supernatant isolated from sow milk. (**A) Physical characteristics observed by Transmission Electron Microscope (TEM), scale bars = 200 nm. **(**B) The size distribution of mEVs is measured by Nanosight. **(**C) The marker protein expression in mEVs and mEVs-removed supernatant. **(**D) The concentration of particles of mEVs and mEVs-removed supernatant. Data are expressed as means ± SEM. *p < 0.05, **p < 0.01, ***p < 0.001; ns, not significant.


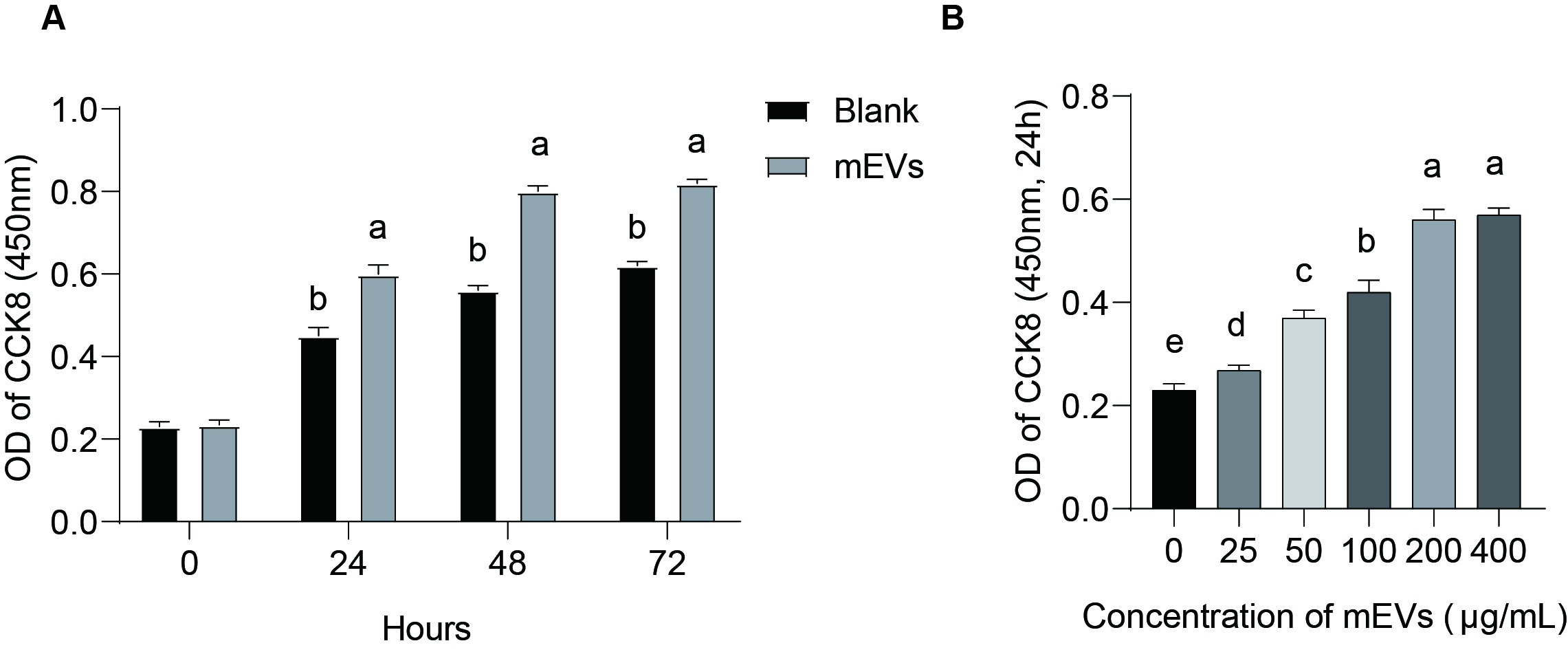


**Figure S2. Gradient experimental design to explore the optimal treatment concentration and treatment time of mEVs in IPEC-J2 cell line (n = 3).** **(**A) The gradient experimental design for optimal treatment time. The spherical test results were not satisfied (p = 0.01), the difference between groups was significant (p = 0.03), and interaction did not exist (time*group = 0.15). The OD of cell culture at each time point was analyzed separately and data are expressed as means ± SD. **(**B) The gradient experimental design for optimal concentration, data are expressed as means ± SEM. Differences in superscript letters for the peer data indicate that the difference is significant (p < 0.05).


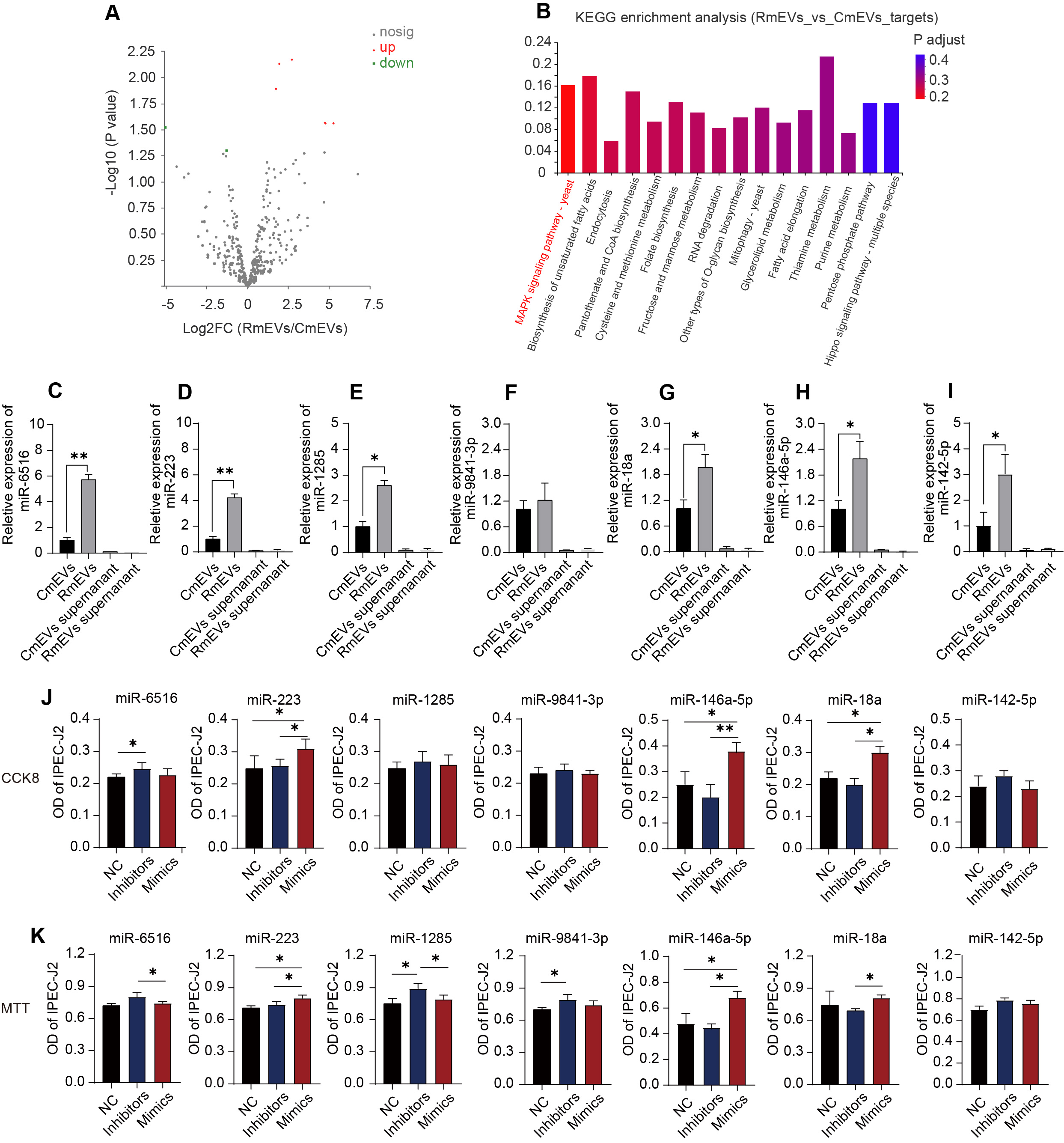


**Figure S3. The miRNA expression profiles in two kinds of mEVs and their effects on the proliferation of IPEC-J2 cells (n = 3).** **(**A) The volcanic map of expression of microRNA between the CmEVs and RmEVs. Red, upregulated in RmEVs. Bule, upregulated in CmEVs. **(**B) KEGG pathway analysis of target genes of miRNAs differently expressed in CmEVs and RmEVs. **(**C-I) The relative expression of miR-6516, miR-223, miR-1285, miR-9841-3p, miR-18a, miR-146a-5p, miR-142-5p in CmEVs, RmEVs, and their supernatants measured by RT-qPCR. **(**J and K) The cell proliferation of IPEC-J2 after transfected by mimics, inhibitors, and NC of miR-6516, miR-223, miR-1285, miR-9841-3p, miR-18a, miR-146a-5p, and miR-142-5p, respectively, then measured by MTT assay. Data are expressed as means ± SEM. *p < 0.05, **p < 0.01.


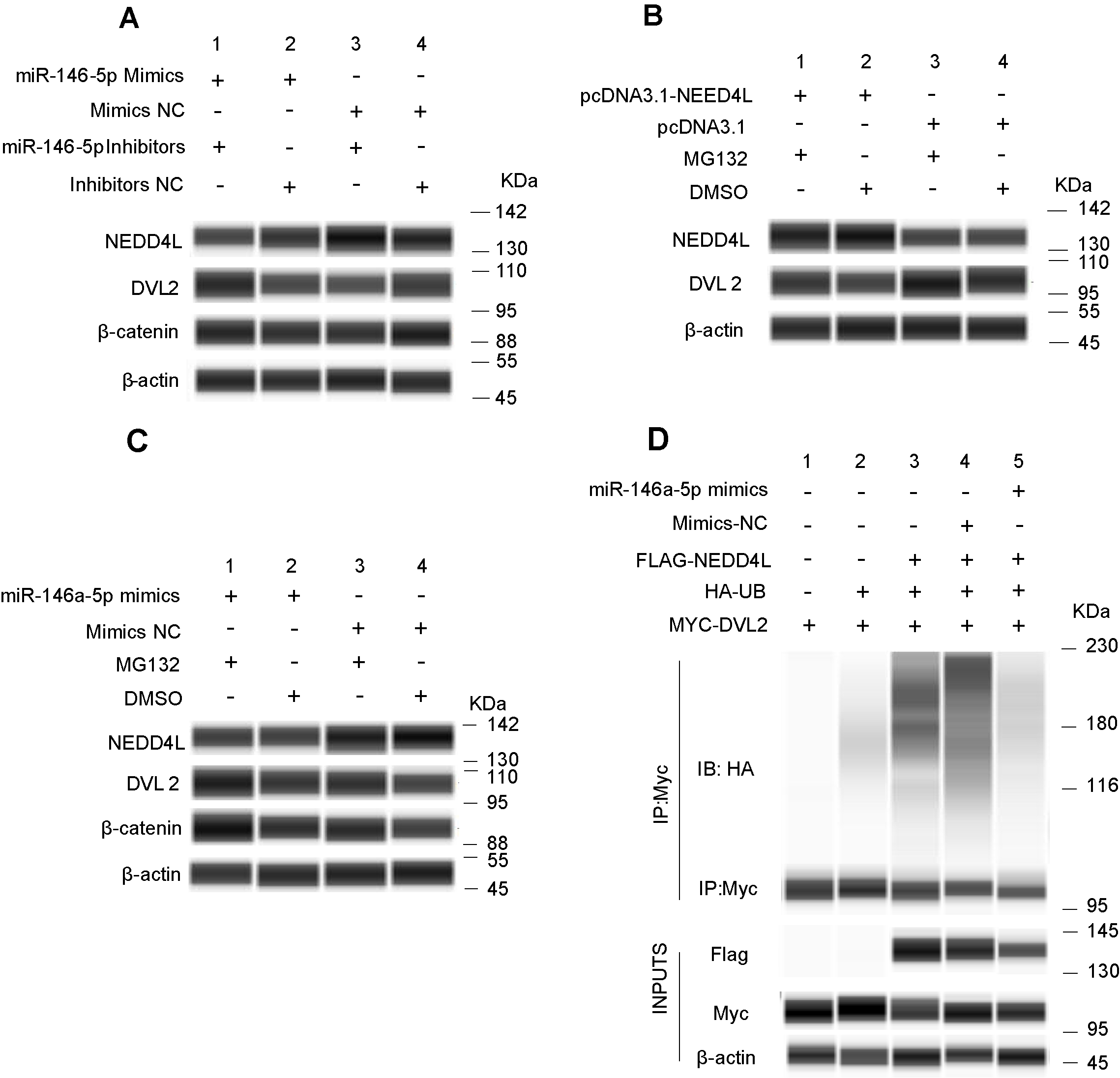


**Figure S4. The high resolution images of Western blot obtained by the WES^TM^ System. (**A) IPEC-J2 cells transfected with miR-146a-5p mimics. (B) IPEC-J2 cells were transfected with pcDNA3.1-NEDD4L or pcDNA3.1-control, then treated with MG132 or DMSO. (C) IPEC-J2 cells were transfected with miR-146a-5p mimics or NC, then treated with MG132 or DMSO. (D) HEK293 cells were transfected with constructs expressing Flag-NEDD4L, HA-Ubiquitin (HA-UB), MYC-DVL2, and miR-146-5p mimics or mimics-NC, followed by anti-MYC IP detection.


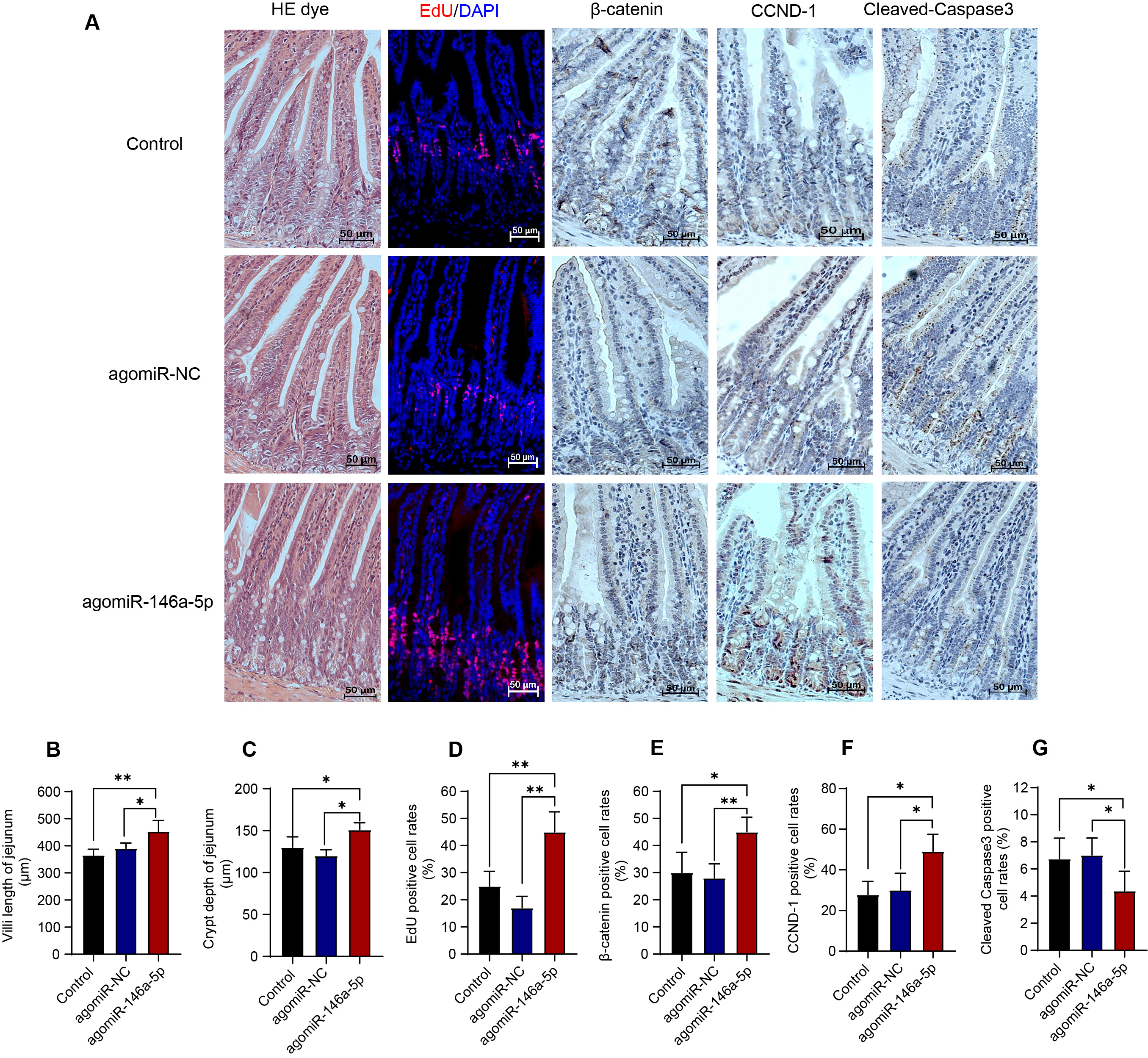


**Figure S5.** **Overexpression of miR-146a-5p increases the proliferation and renewal of intestinal cells in mice model (n = 8).** **(**A) Representative images of jejunum stained for HE, EdU, β-catenin, CCND1, and Cleaved-Caspase 3. Scale bars: 50 µm. **(**B and C) The villi length and crypt depth of jejunum. **(**D-G) The ratio of positive cells in jejunum for EdU, β-catenin, CCND1, and Cleaved-Caspase3. Data are expressed as means ± SEM. *p < 0.05, **p < 0.01.
